# Supplementary material for: A phase transformable ultrastable titanium-carboxylate framework for photoconduction
Source: Nat Commun. 2018 Apr 25;9:1660. doi: 10.1038/s41467-018-04034-w (PMC5916937; doi:10.1038/s41467-018-04034-w)
Supplement: Supplementary file 1 — Supplementary Information [file 41467_2018_4034_MOESM1_ESM.pdf]

Supplementary Information

A phase transformable ultrastable titanium-carboxylate framework for photoconduction

Sujing Wang et al.

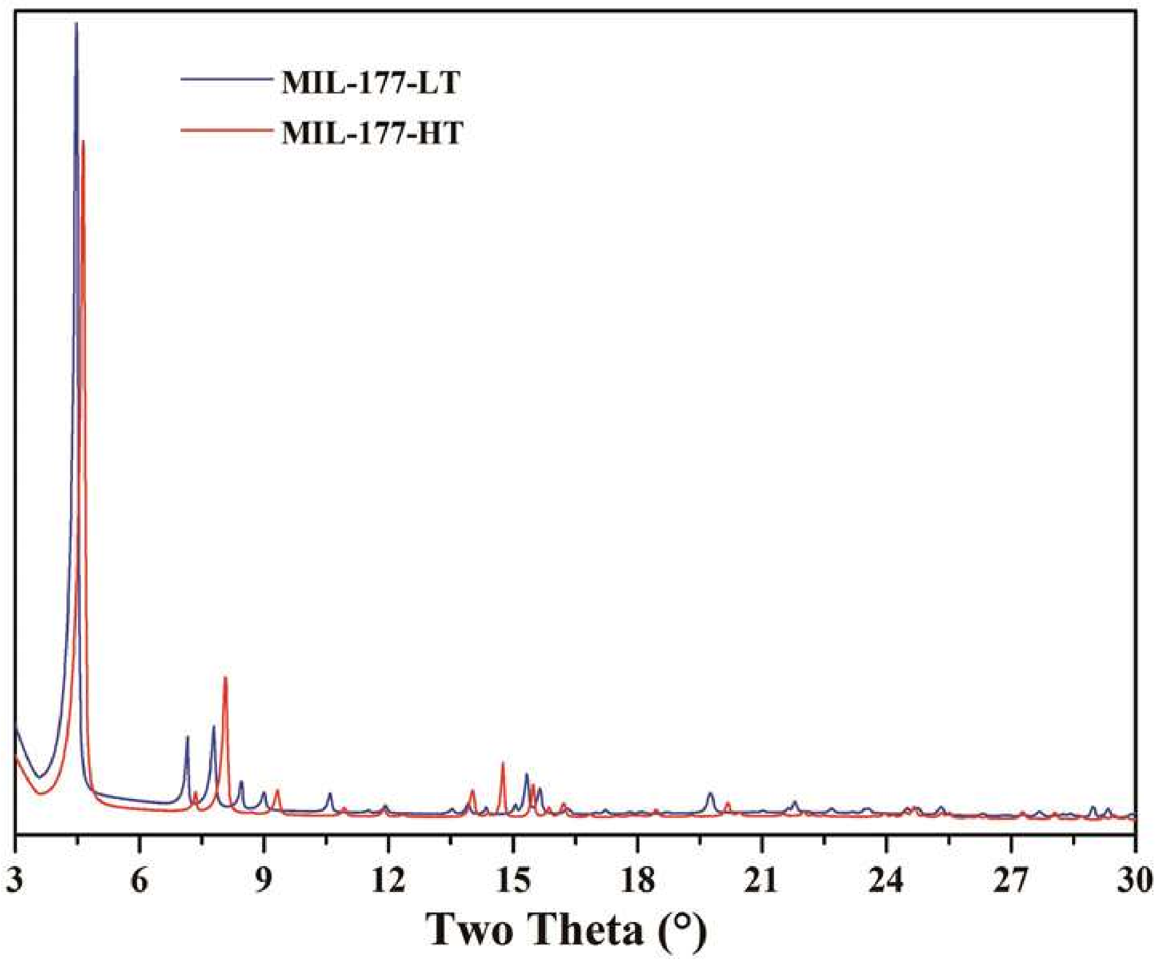

Supplementary Figure 1. PXRD patterns of MIL-177-LT (blue) and MIL-177-HT (red).

Both MIL-177-LT and MIL-177-HT samples were grinded into fine powder and sealed in glass capillaries with an inner diameter of 0.3 mm. PXRD patterns were collected on a Bruker D8 diffractometer at room temperature for 72 hours in a 2θ range from 3-80 º. It is clear that the PXRD peaks of the MIL-177-HT structure have a shift to higher 2θ angle range, corresponding to its decreased unit cell parameters compared to that of the MIL-177-LT structure.

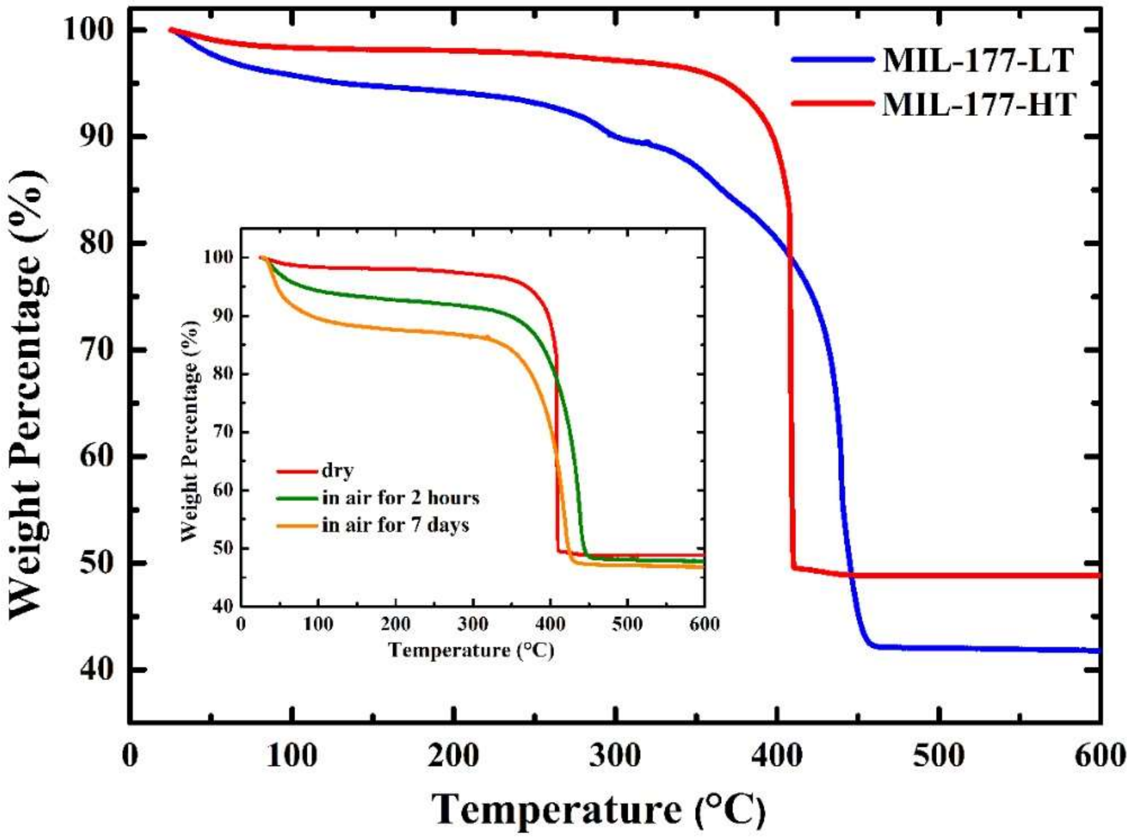

Supplementary Figure 2 TGA curves of MIL-177-LT (blue) and MIL-177-HT (red). The inserted part showed the guest water content absorbed in HT porosity upon exposure time in air.

For the MIL-177-LT as-made sample (in blue), weight loss upon heating was found in the temperature range of 25 °C to around 280 °C, which correspond to free solvent molecules (formic acid and water). Afterwards, another step of weight loss took place in the temperature range of 280 °C to 300 °C, which is in good agreement with the removal of coordinated formate species (terminal formates facing the channel running along the *c*-axis and bridging formates which connect the adjacent Ti_{12}-oxocluster SBUs along the *c*-axis respectively). The departure of the organic linker and related MOF structure decomposition took place when the temperature was above 350 °C, resulting in TiO_2 as the final inorganic residue. In contrast the MIL-177-HT compound (in red) shows very limited weight loss from the beginning until 350 °C with a wide

range plateau suggesting the accessible void cavity in the MOF structure. As shown in the above inserted figure, MIL-177-HT adsorbed water molecules slowly when it was taken out from the calcination oven and exposed in air. 5% of guest water molecules were detected when the dry sample was let in air for 2 hours (in green) while 13% of water was determined for the sample stayed in air for 7 days (orange curve). The relatively much slower adsorption of water of MIL-177-HT in air compared to some reported hydrophilic MOFs, such as MIL-53, NH_2-MIL-125 and MIL-160, evidenced the hydrophobic environment of the porosity in the MOF structure.

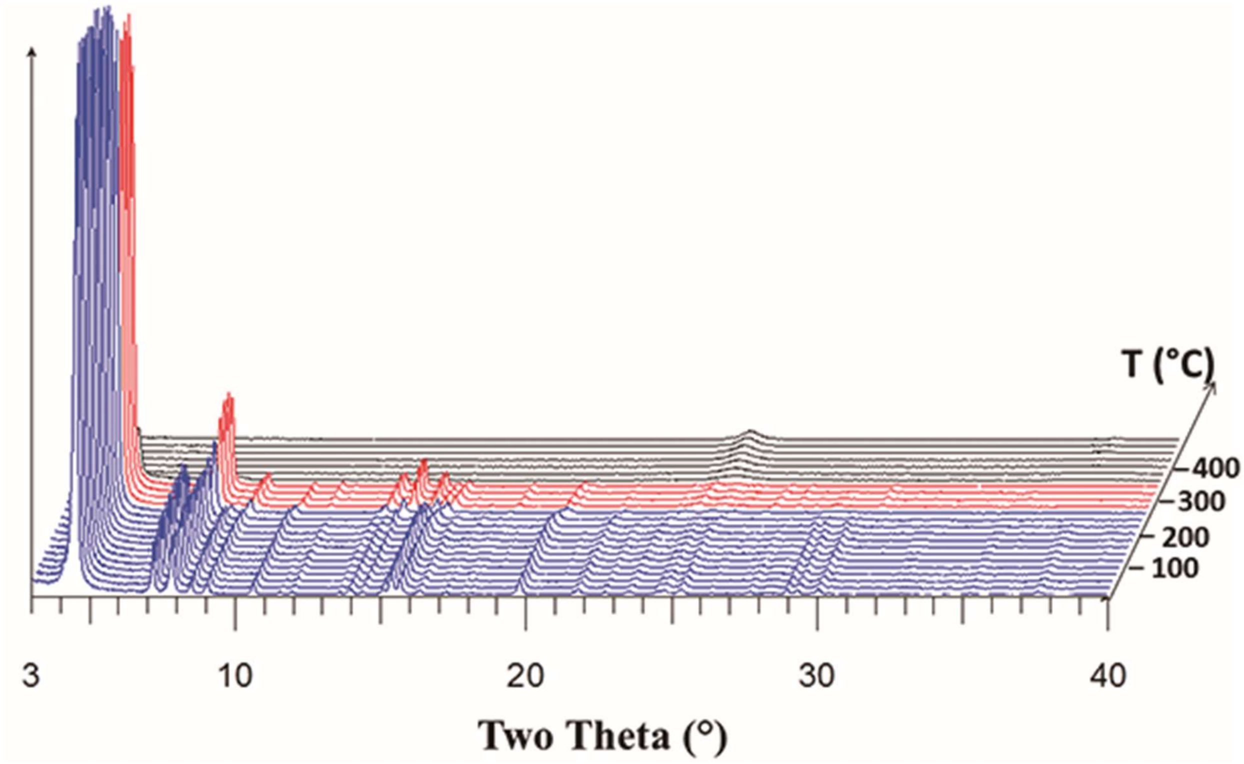

Supplementary Figure 3 Thermal evolution of the powder X-ray diffraction data of the MIL-177.

As shown in Supplementary Figure 3, PXRD patterns do not show notable changes below 275 °C, suggesting that departure of solvent molecules in the MIL-177-LT structure occurs without important structural changes. Afterwards the phase transformation to MIL-177-HT structure took place and was completed slowly upon heating. The MIL-177-HT structure has a thermal stability up to around 350 °C and then TiO_2 crystallizes with decomposition of the MOF framework.

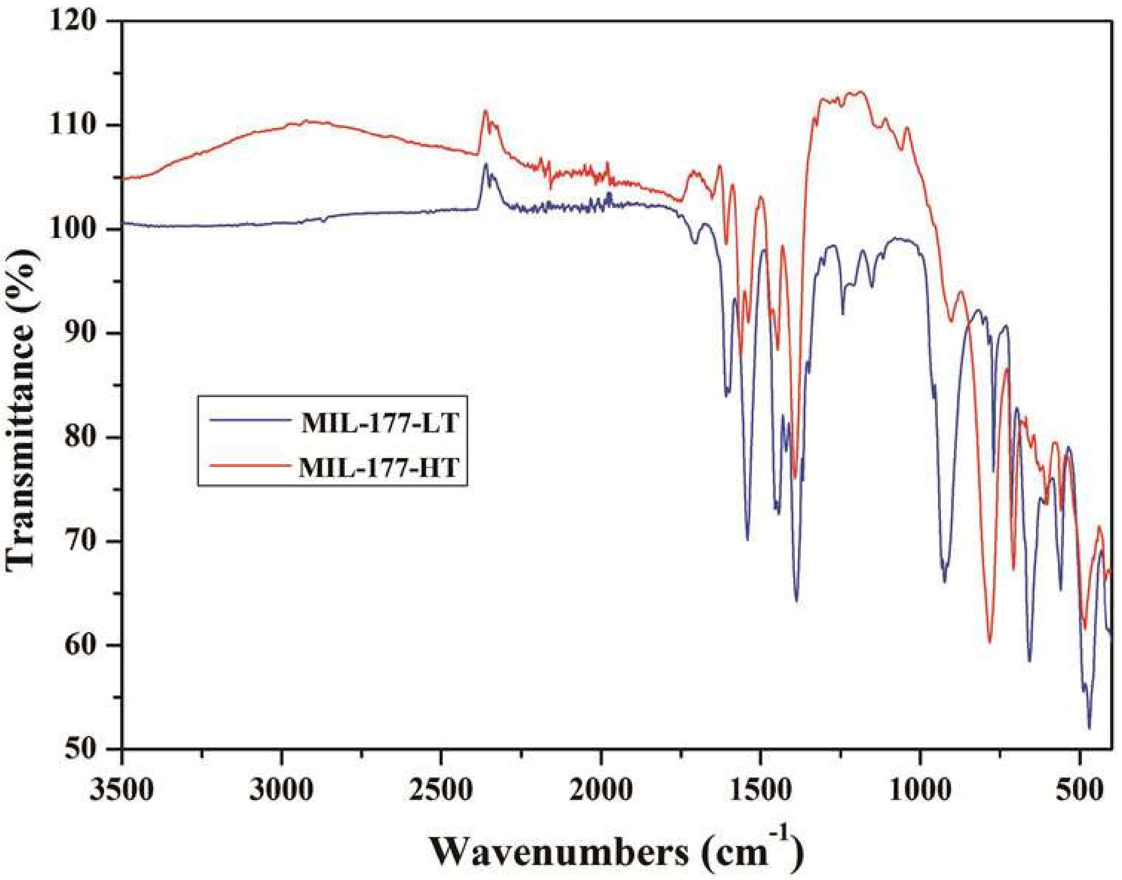

Supplementary Figure 4 IR spectra of MIL-177-LT (blue) and MIL-177-HT (red).

As shown in the above figure, the peaks in the range from 1700 to 1200 cm^{-1} for both MIL-177-LT and MIL-177-HT are very close despite slight difference of relative transmittances of a few peaks, suggesting the similarity of chemical environment and connection fashion for the organic linker in both structures. However, the lower wavenumber range from 1000 to 500 cm^{-1} is relatively more complicated. Notable differences between the two samples could be observed possibly due to the inner SBU connection arrangement and change of the oxo groups. It notes worthy that both MIL-177-LT and MIL-177-HT structures have quite hydrophobic voids as almost no peak in the region of 3000-3500 cm^{-1} could be found which normally corresponds to hydroxyl groups or water molecules.

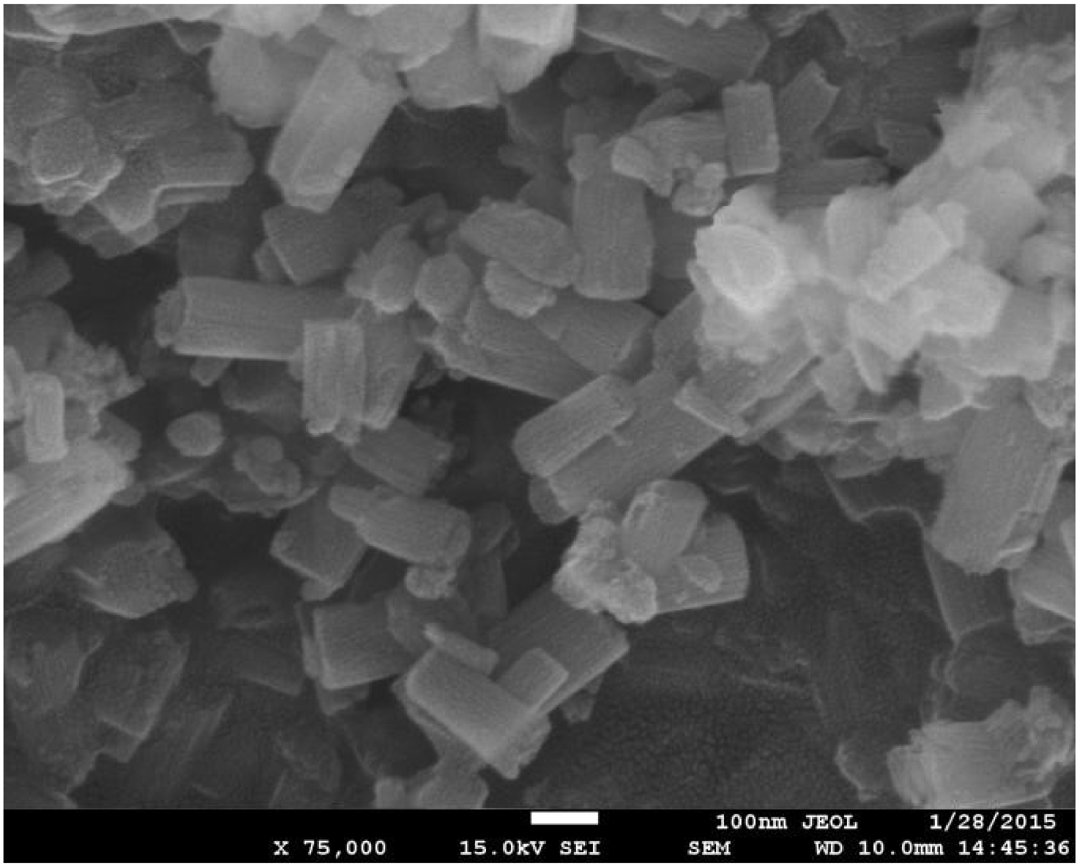

Supplementary Figure 5 SEM image MIL-177-HT.

From the above figure, it is found that the microcrystals of MIL-177-HT have a hexagonal rod morphology which corresponds well with their *P*6/mmm hexagonal space group crystal structure. The thermal treatment for the MIL-177-HT product preparation did not show notable influence on its particle morphology. Noteworthy, the MIL-177 product is already in nano-material scale which is really helpful for following applications in different aspects.

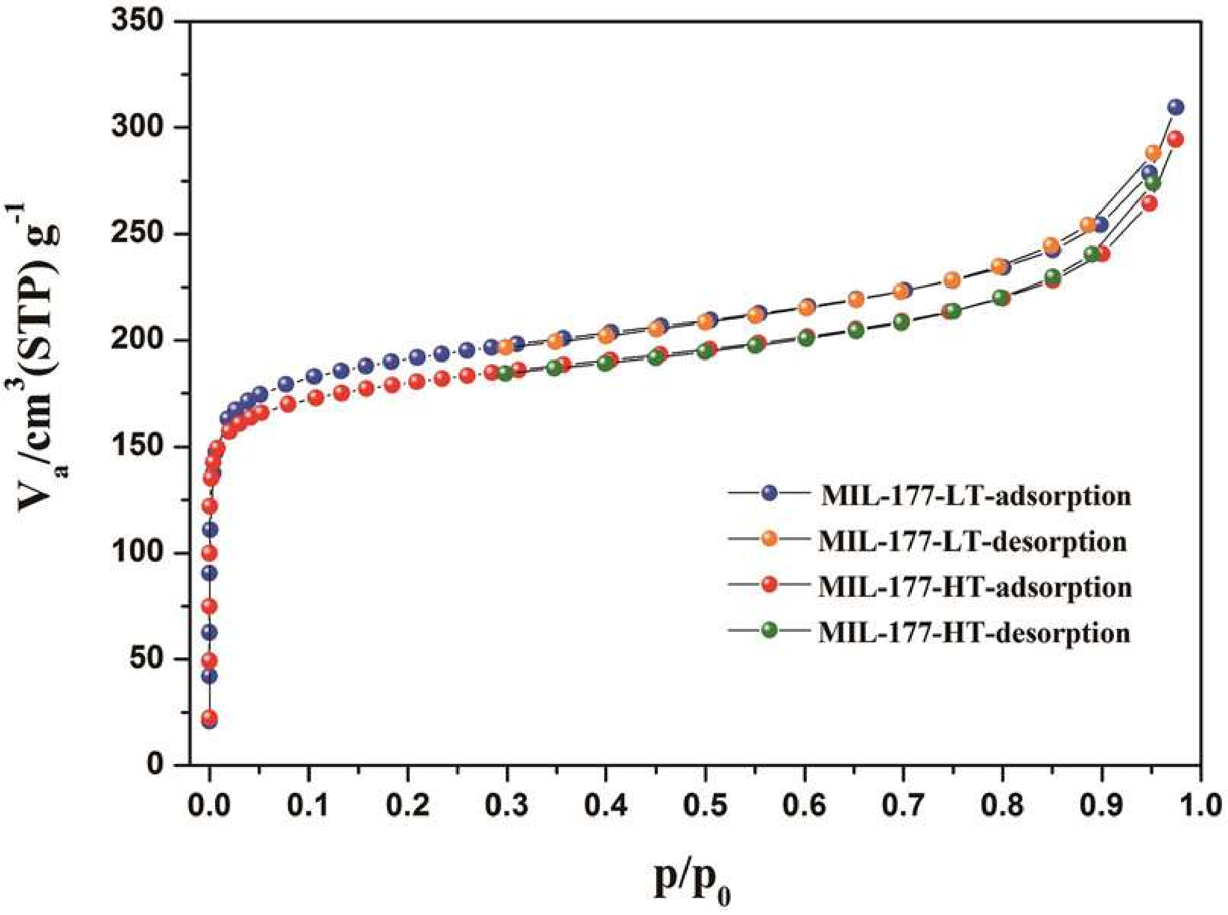

Supplementary Figure 6 Nitrogen sorption isotherms of MIL-177-LT (blue) and MIL-177-HT (red) at 77K.

The MIL-177-LT and MIL-177-HT samples were directly activated thermally at 230 ºC and 120 ºC separately for 20 hours under vacuum before the nitrogen adsorption at 77K were carried out. As MIL-177-HT was obtained by heating MIL-177-LT compound at 280 °C during which all the guest molecules have already been evacuated completely, activation at 120 ºC under vacuum is enough to remove the re-adsorbed water in air. According to the nitrogen sorption isotherms showing in the above figure, the Brunauer-Emmett-Teller (BET) surface area, Langmuir surface area and total pore volume (p/p_0 = 0.990) of MIL-177-LT were calculated to be 730(10) m^2/g, 830(4) m^2/g and 0.47(8) cm^3/g respectively. For the MIL-177-HT sample, after removal of

formates and rearrangement of the framework connection, the major channel running along the *c*-axis contracted a little bit, which corresponds well with the decreased value of the BET surface area, Langmuir surface area and total pore volume ($p/p_0 = 0.990$) of 690(10) m^2/g, 780(8) m^2/g and 0.45(5) cm^3/g respectively.

The theoretical N_2-accessible surface area was calculated based on the geometric topology of MIL-177 and a Monte Carlo integration technique where the center of mass of the probe molecule with hard sphere is "rolled" over the framework surface. In this method, a nitrogen-sized (3.681 Å) probe molecule is randomly inserted around each framework atom of the adsorbent and the fraction of the probe molecules without overlapping with the other framework atoms is then used to calculate the accessible surface area. The Lennard-Jones size parameters of the framework atoms were also taken from DREIDING force field except that the size parameter for Zr atom was taken from UFF.

The accessible volume was also calculated using a similar geometric method as mentioned above which consists of using a probe molecule with a diameter of 0 Å to determine the volume of the porous solid that is not occupied by the atoms of the framework. One obtains what is usually called the "free volume".

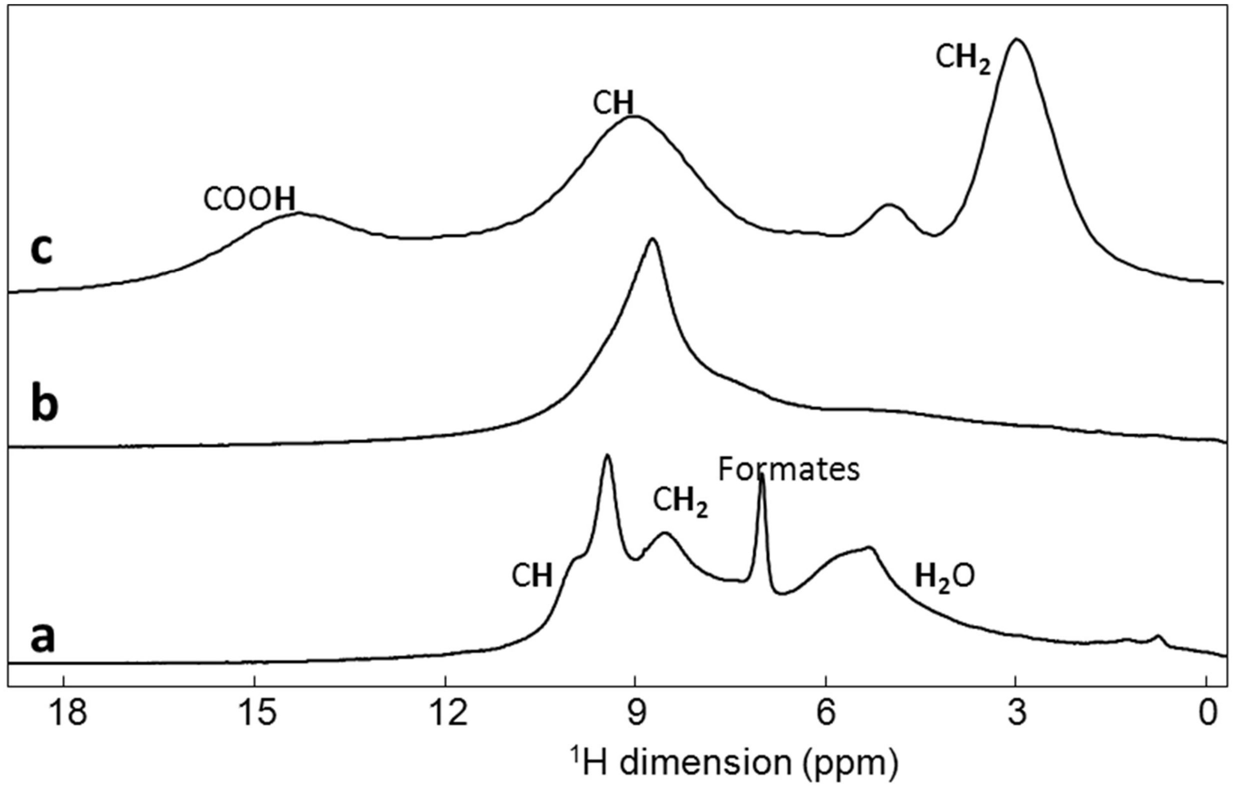

Supplementary Figure 7. ^{1}H MAS NMR spectra of **a** MIL-177-LT and **b** MIL-177-HT, showing the absence of the formates in the MIL-177-HT structure. **c** spectrum of the initial mdip free linker. The absence of the acidic proton resonance in the final MOFs confirms the connection of all four carboxylates to the titanium ions. The lines are labeled.

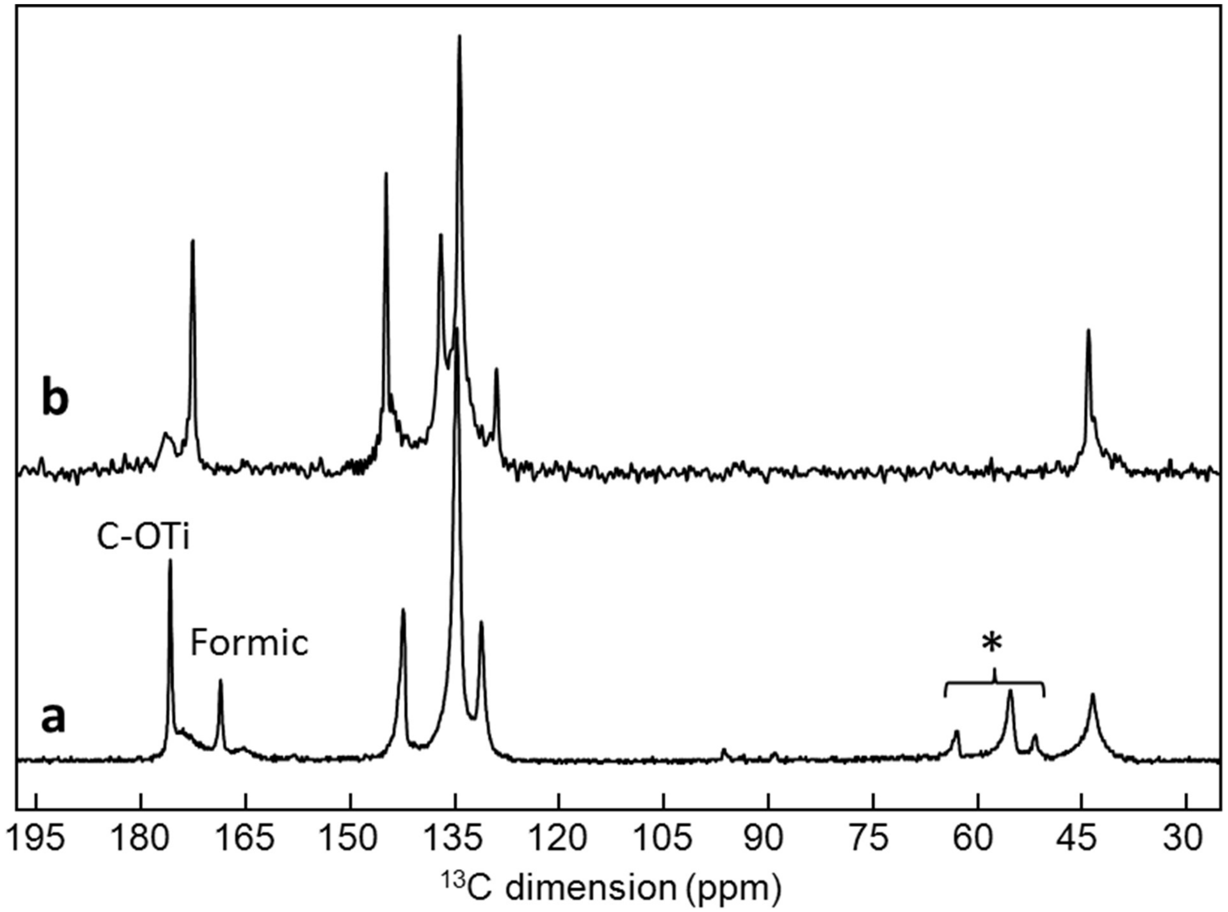

Supplementary Figure 8 Quantitative ^{13}C MC-CPMAS NMR spectra of **a** MIL-177-LT and **b** MIL-177-HT: The lines are assigned based on the relative line intensity and the correlation of the 2D ^{1}H-^{13}C MAS NMR spectrum. Stars represent spinning sidebands. In the MIL-177-HT structure, the formats are no longer present.

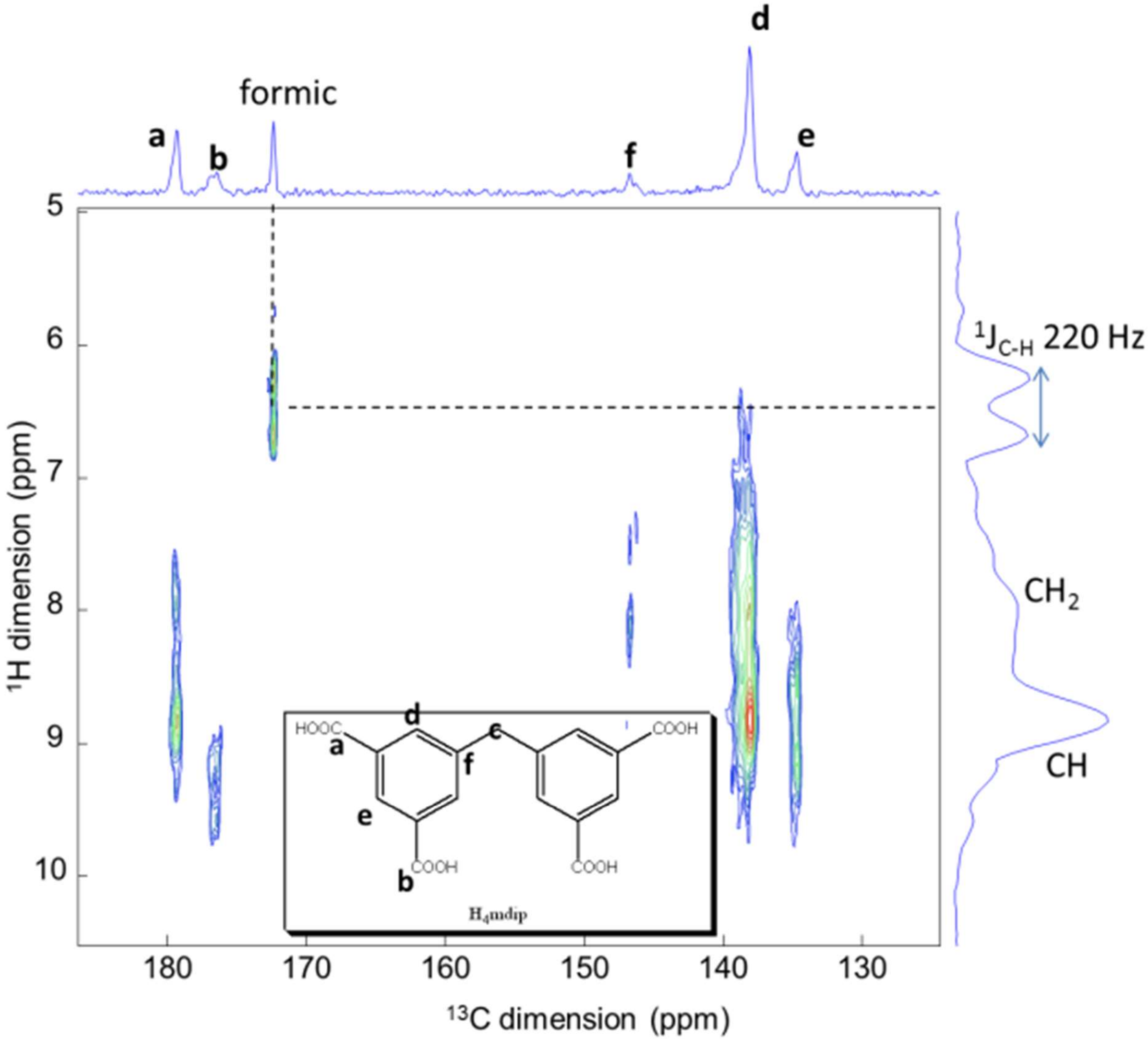

Supplementary Figure 9 ^{1}H-^{13}C 2D MAS NMR correlation spectrum of MIL-177-LT. The ^{13}C relative line intensity of the MC-CPMAS NMR spectrum and the correlation pattern allow assigning the ^{13}C resonances, and confirm the presence of the formate species.

The $^1H \rightarrow ^{13}C$ multiple-contact cross-polarization (MC-CP)[1] experiment was applied, which allows obtaining quantitative ^{13}C CP spectra in protonated MOFs[2]. 10 CP blocks of 1000 μs each (total contact time of 10.1 ms) with repolarization periods of 1 s were applied. The $^1H \rightarrow ^{13}C$ CP conditions used radiofrequency (RF) fields of 60 and 50 kHz on ^{1}H and ^{13}C, respectively. 256 to

1024 transients were co-added with 4 s recycle delay. ^{1}H SPINAL-64 decoupling[3] was applied. The ^{1}H NMR spectra were recorded at MAS frequency of 30 kHz, using a 90°-180°-90° Hahn-echo sequence. The 90° pulse length was 2.5 μs, and the inter-pulse delay was synchronized with one rotor period. The recycle delay was set to 5 s and 16 transients were recorded for each sample. The ^{1}H→^{13}C 2D CP-heteronuclear correlation (CP-HETCOR) NMR spectrum was recorded at MAS 30 kHz, using a contact times of 3 ms. 120 t_1 slices with 1024 transients each were co-added (recycle delay of 2 s). The spectra were analyzed using the dmfit software[4]. The ^{1}H and ^{13}C chemical shifts were referenced to proton and carbon signals in TMS.

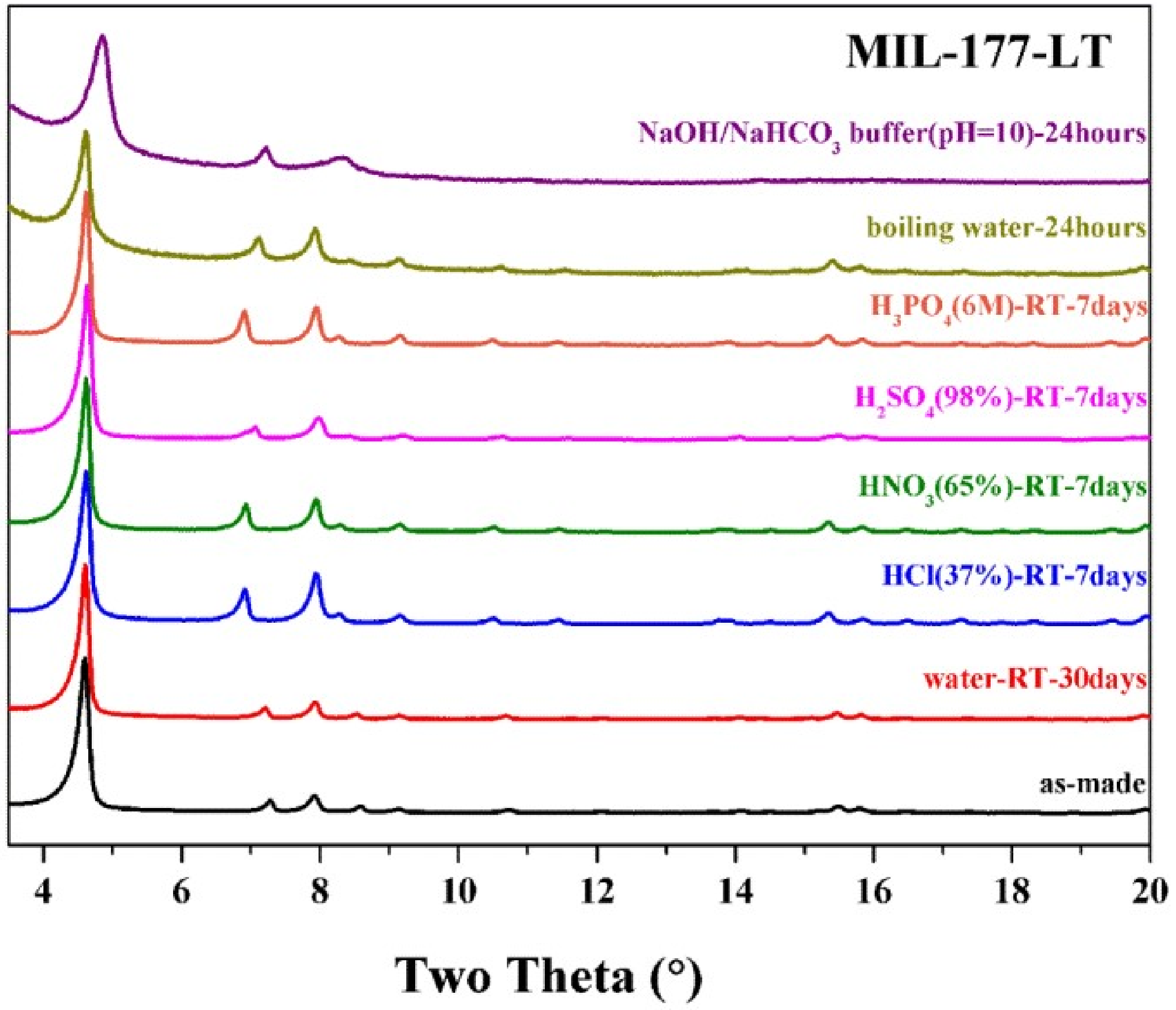

Supplementary Figure 10 Chemical stability test results for MIL-177-LT as-made sample

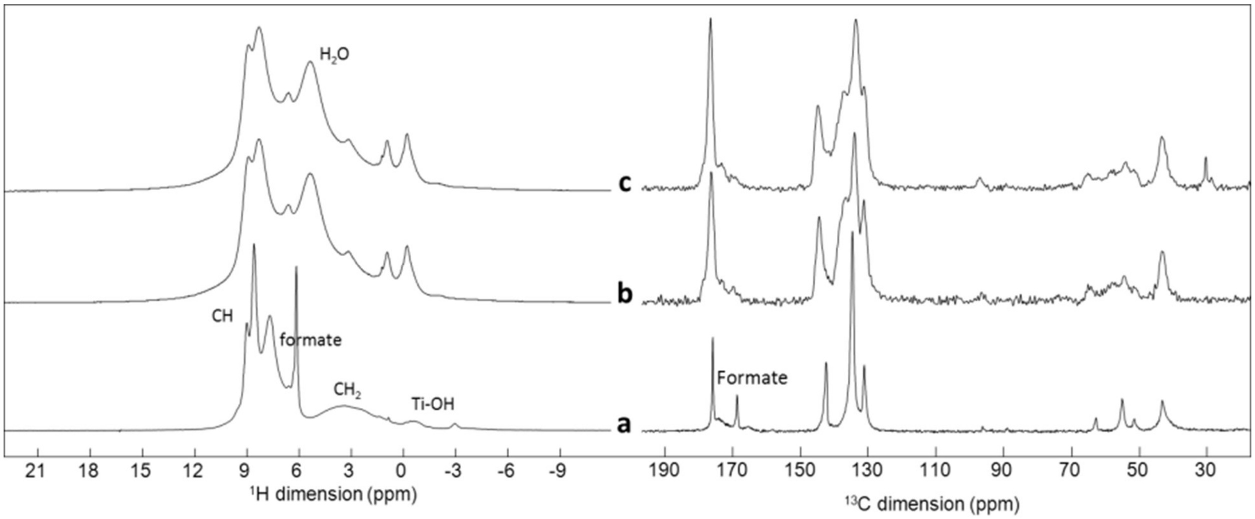

Supplementary Figure 11 ^{1}H (left) and ^{13}C CP (right) MAS NMR spectra of MIL-177-LT **a** as-made, **b** treated with H_3PO_4 and **c** H_2SO_4 concentrated solutions. In the treated samples, the formate species are removed, but the framework stays intact (no shift of the ~ 180 ppm resonances that correspond to the C-O-Ti carbon atoms).

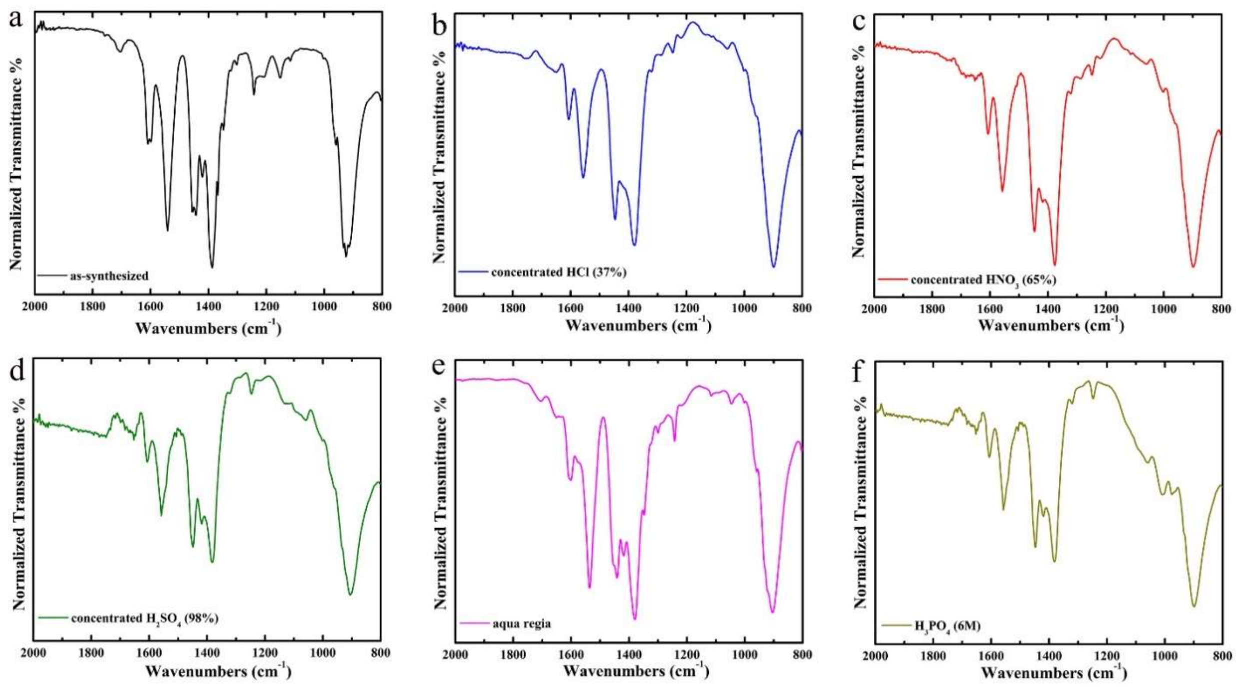

Supplementary Figure 12 FT-IR spectra of MIL-177-LT samples before and after the treatment in various acids.

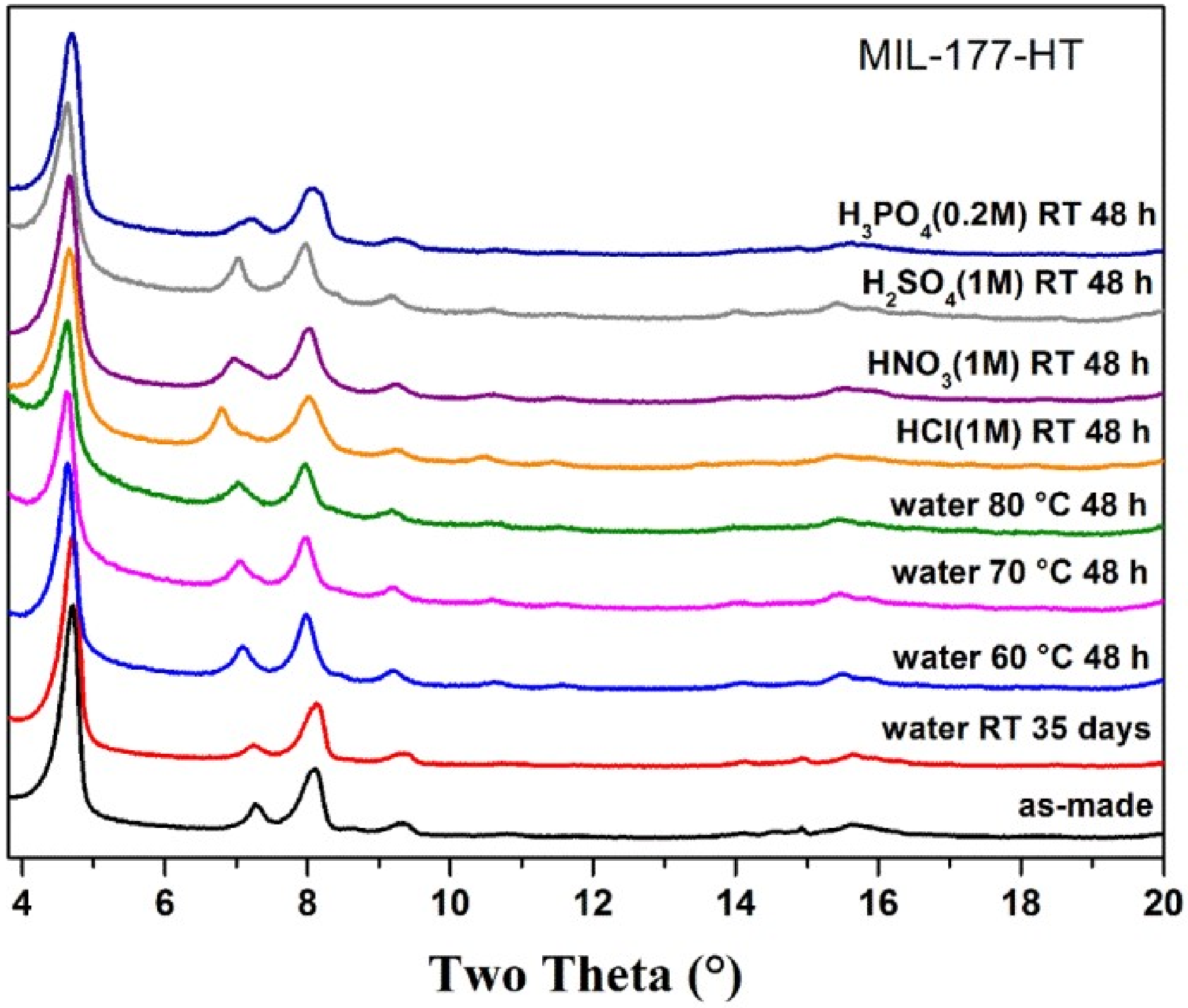

Supplementary Figure 13 Chemical stability test results for MIL-177-HT sample.

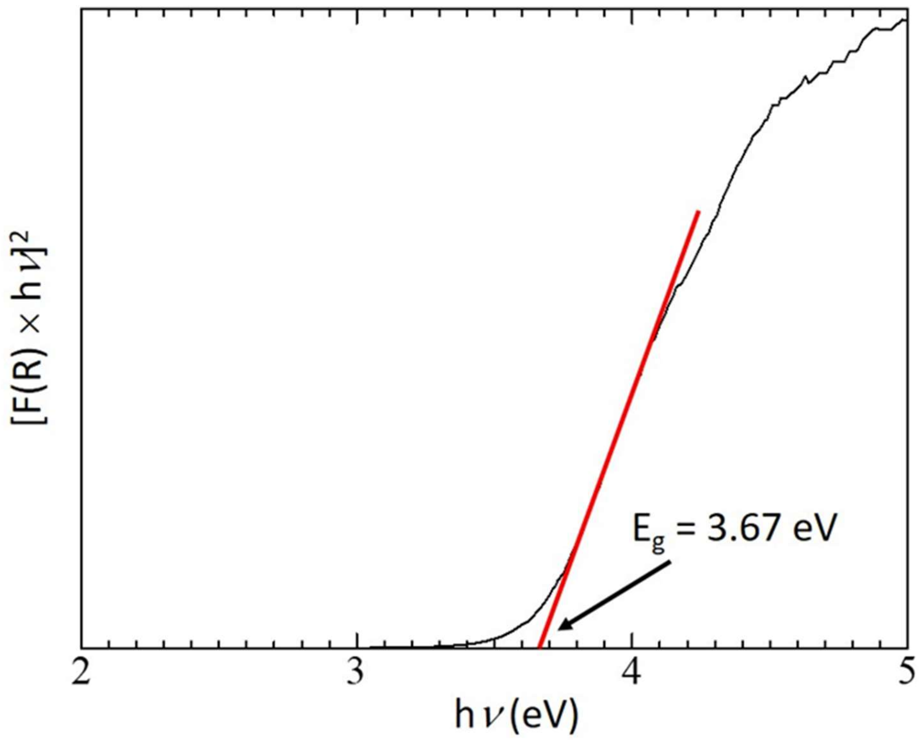

Supplementary Figure 14. Tauc plot ($[F(R) \times h\nu]^2$ vs $h\nu$) for bandgap transition for MIL-177-HT

The band gap was determined from diffuse reflectance data using the Kubelka−Munk (KM) method, which is given by the following equation:

$$\frac{K}{S} = F(R) = \frac{(1-R)^2}{2R}$$

Where R is the reflectance, F(R) is the KM function, and K and S are the absorption and scattering coefficients, respectively.

For allowed direct transition evidenced by analysis of the band structure (Fig. S16), the optical band gap for MIL-177-HT is determined by preparing a Tauc Plot ($[F(R) \times h\nu]^2$ vs $h\nu$).[5] Extrapolation of this line to the photon energy axis yields a band gap of 3.67eV.

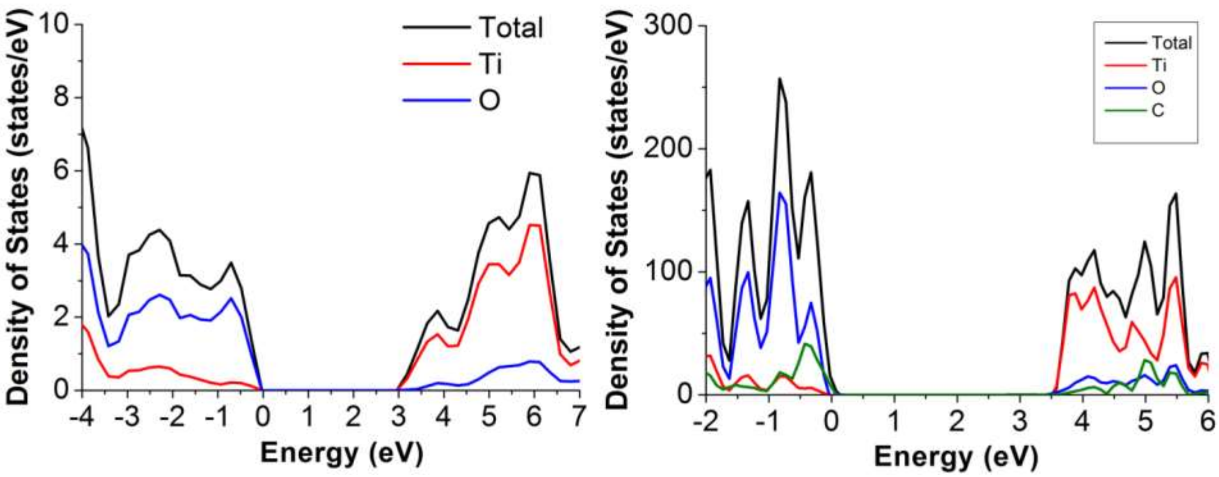

Supplementary Figure 15. Total and partial PDOS of (left) TiO_2 anatase and (right) MIL-177-HT calculated at the PBEsol-level to determine the contribution of the different orbitals whereas the reported band gap is calculated using HSE-06 functional.

The final structure model of MIL-177-HT issued from the joint experimental-modelling approach was further optimized with a view to the electronic structure calculations. The initial optimization was carried out using the PBE-sol functional. The subsequent optimization used the HSE06 hybrid DFT-Hartree Fock functional[6], which has been shown to be accurate for the calculation of band gaps and HOMO-LUMO gaps[7, 8, 9]. In this functional, 25% of the exchange part is treated using Hartree-Fock. The electronic band structures and density of states (DOS) were calculated using the VASP planewave code[10], using the same strategy as Hendon et al[11] (PBEsol functional and PAW scalar relativistic pseudopotentials[12], and an energy cut-off of 500 eV).

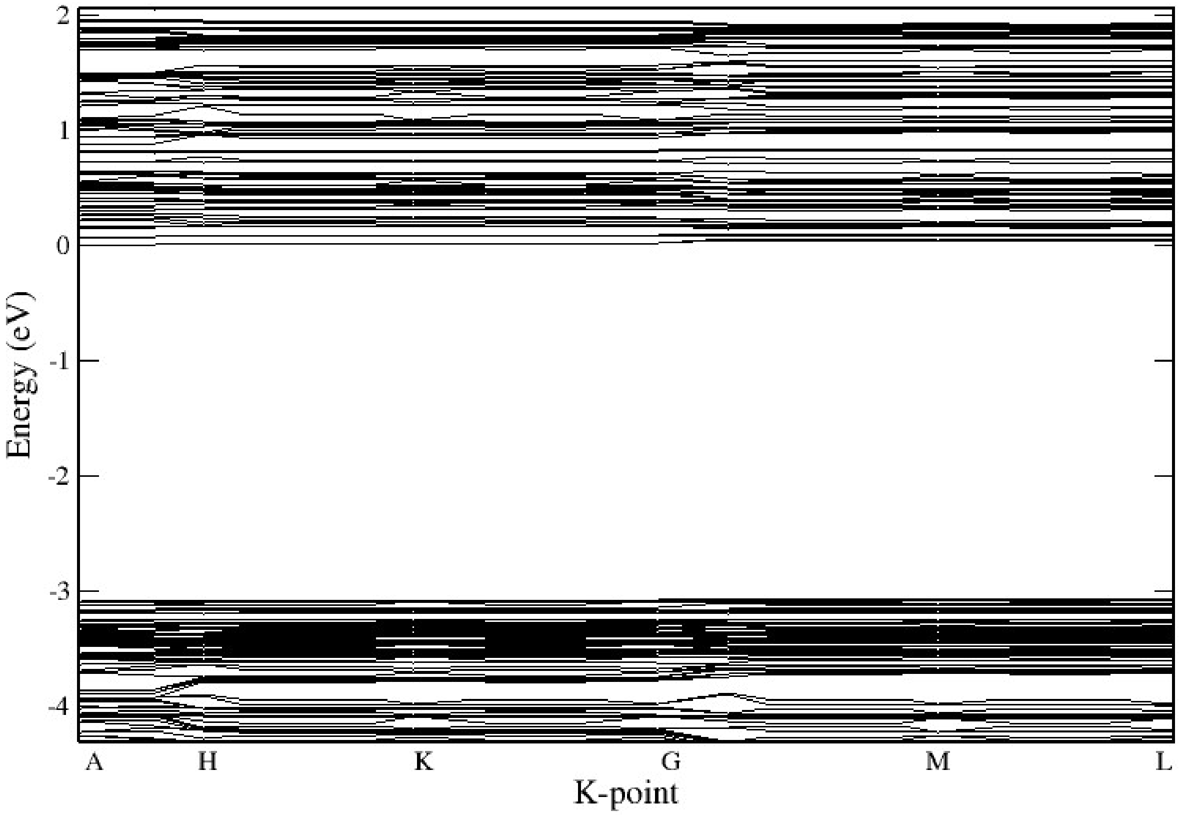

Supplementary Figure 16. Band structure of MIL-177-HT.

The fermi levels of MIL-177-HT and TiO_2 anatase are as follows -3.1 eV and -2.6 eV respectively. The band structures of MOFs mostly feature many blocks, due to the large unit cells and the huge number of bands. They are therefore difficult to glean information from, and it is more useful to analyze the PDOS. This was seen to be the case when we calculated the band structure of MIL-177 HT. This explains why the analysis provided in the paper mostly focused on the PDOS rather than the band structure.

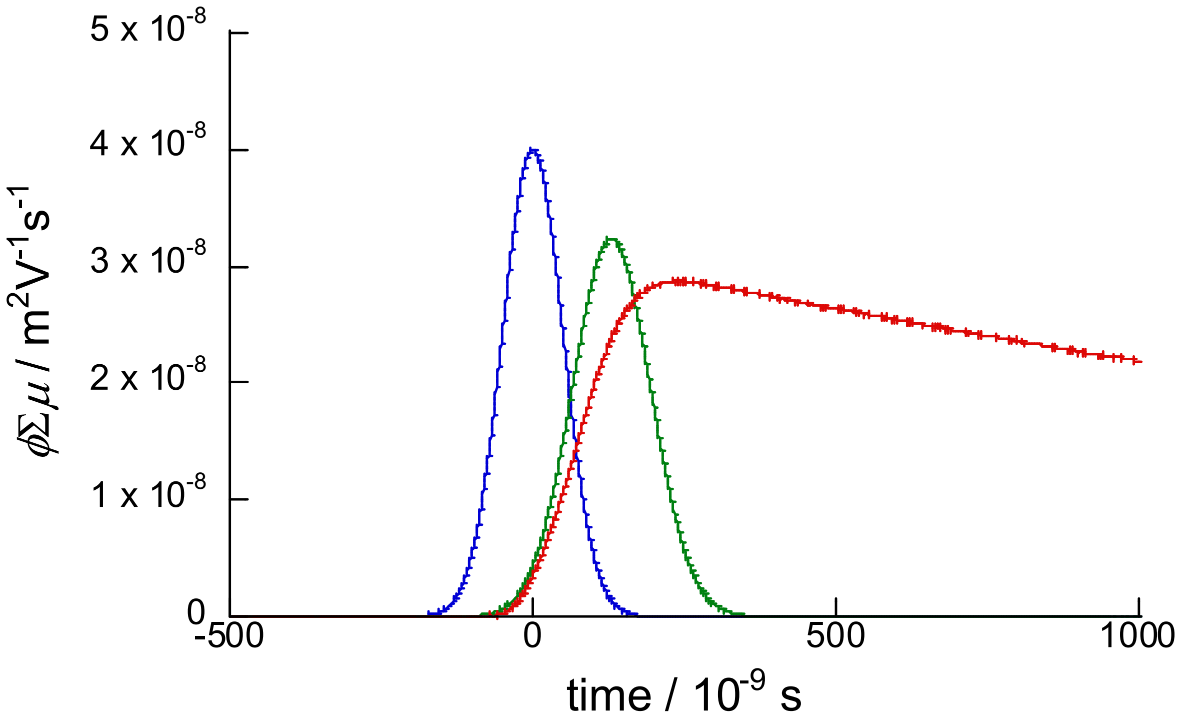

Supplementary Figure 17. Kinetic trace of photoconductivity observed in MIL-177-HT(red) upon exposure to 266 nm pulses of Forth Harmonic Generation from a Spectra-Physics INGI-HG Nd:YAG laser (Fundamental Pulse Duration of 5-8 ns, the enlarged view of figure 3 in the manuscript around the excitation timing.). Green and blue lines are the Gaussian fitting functions for conductivity signal evolution (FWHM = 180 ns) and the Gaussian one deconvoluted from the signal rising (FWHM = 65 ns).

The estimates of quantum efficiency in photo-carrier generation upon excitation at 266 nm laser pulses are presumed as $\phi = 1$ in the present case, because the photon energy of 266 nm pulses are apparently exceeding the optical band gap of MIL-177-HT. The high enough photon energy produced electron-hole pairs quantitatively within the excitation light pulses, thus the estimate of $\phi = 1$ leads the lowest limit of electron mobility in MIL-177-HT.

At first, the conductivity signal of MIL-177-HT in Figure 3 in the main text was evolved after laser pulse excitation as shown evidently in the Figure S18. The signal evolution was fitted by the

Gaussian function with the decay rate of 4.5×10^{-5} s^{-1} ($\tau_{1/2}$ = 2.2 μs as in the manuscript), giving the standard deviation of the function as 76 ns. This is far longer than both the time duration of an excitation light pulse (t < 3 ns for FHG from fundamental light pulses with the duration of 5-8 ns) and the response time of the resonant cavity used in the present set of apparatus with the Q-value of 2200. The time constant (τ_C) of the cavity can be estimated roughly by,

$$\tau_C = \frac{2\pi}{\omega}(Q)^{1/2}$$

where ω is the angular frequency of microwave used in the present system (9.1 GHz). The derived value of τ_C was $\tau_C \sim 50$ ns, thus to address the peak conductivity soon after an excitation by the short-enough pulses, the deconvolution by another Gaussian function with the standard deviation of 50 ns was carried out. Eventually, as seen in the figure, the maximum value of $\phi\Sigma\mu$ was derived as 4×10^{-4} $cm^2V^{-1}s^{-1}$, and this is the case of the value of mobility given in the manuscript. Details of the set of apparatus were described elsewhere[13].

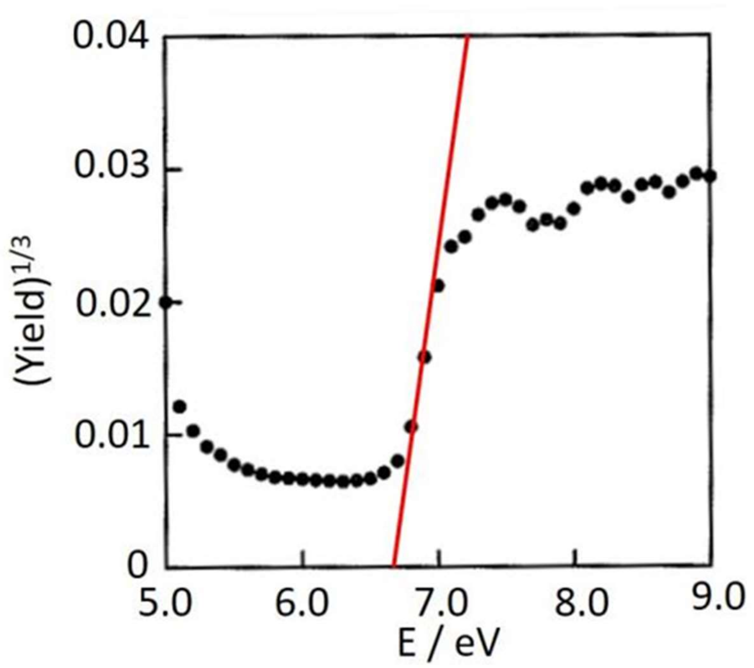

Supplementary Figure 18. Ultraviolet photoelectron spectroscopy (UPS) of MIL-177-HT

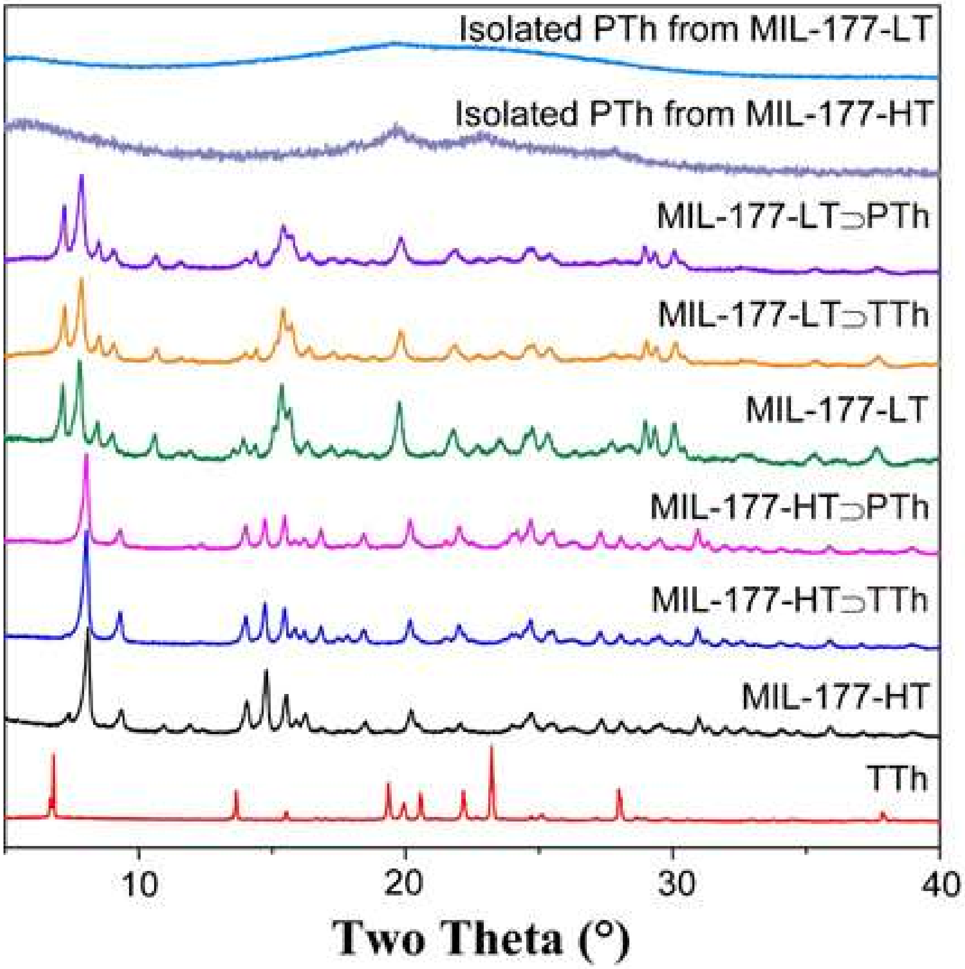

Supplementary Figure 19. PXRD patterns of MIL-177, TTh, MIL-177⊃TTh, MIL-177⊃PTh, and PTh isolated from MIL-177.

The absence of diffraction characteristic of crystalline TTh demonstrated that there was no leakage of the monomer from the nano-channels of MIL-177. PXRD patterns of MIL-177⊃PTh indicated that the crystal structures of MIL-177 were maintained during the polymerization and dedoping processes. The obvious changes in the relative peak intensities were detected when compared to those of the only hosts, and these changes of the intensity ratios were attributed to a variation of electron density in the pores[14]. Consistently, the same change of the relative intensities was observed after the encapsulation and polymerization of TTh in the pores of MIL-177.

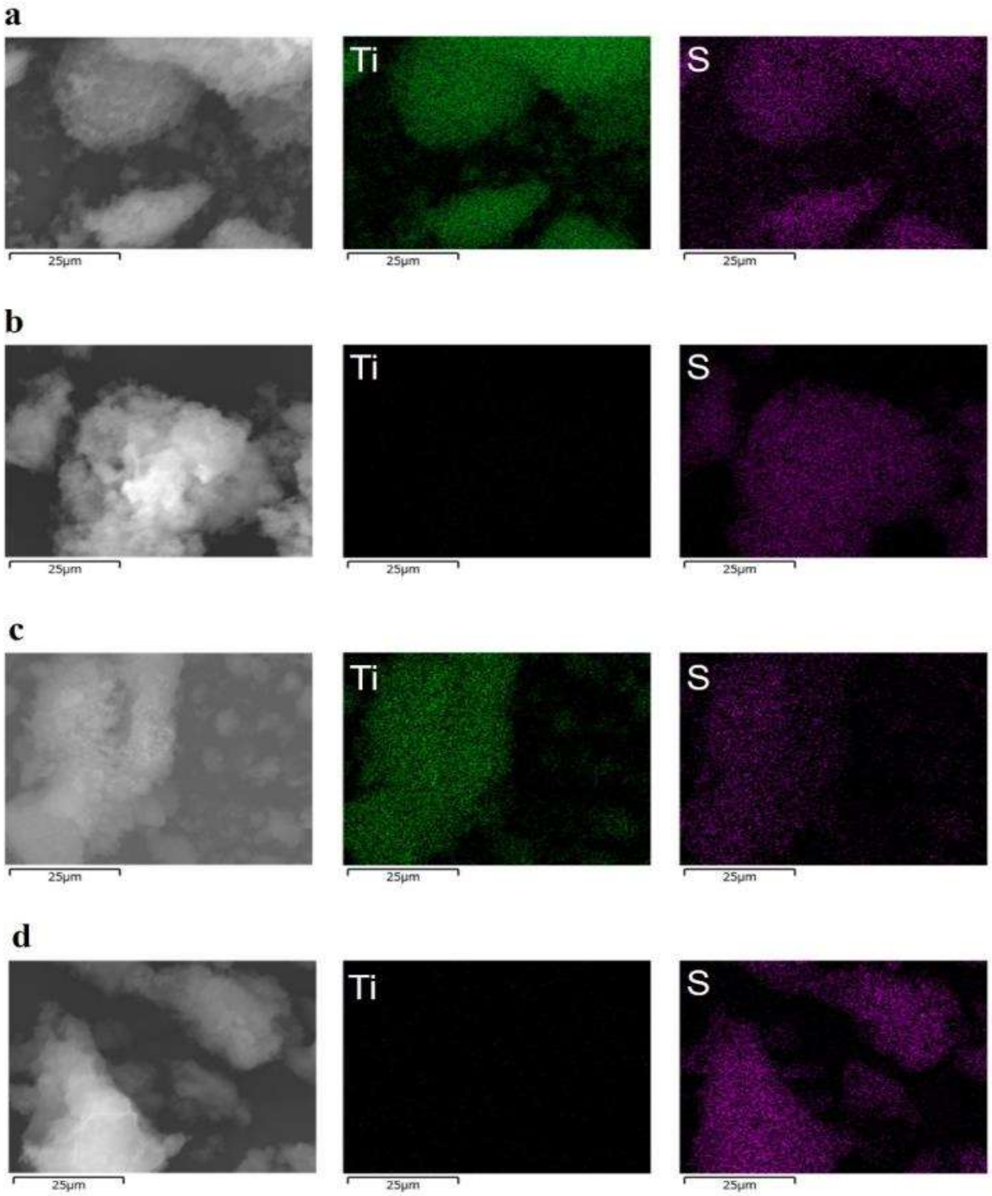

Supplementary Figure 20. SEM-EDS elemental (Ti and S) mapping of **a** MIL-177-HT⊃PTh, **b** isolated PTh from MIL-177-HT, **c** MIL-177-LT⊃PTh, and **d** isolated PTh from MIL-177-LT.

SEM-EDS elemental mapping analysis for MIL-177⊃PTh confirmed the homogeneous distribution of S atoms in the MOF crystals, indicating homogeneous dispersion of the polymer chains in the host nanochannels. Ti mapping assured the complete removal of MOF architecture during polymer recovery.

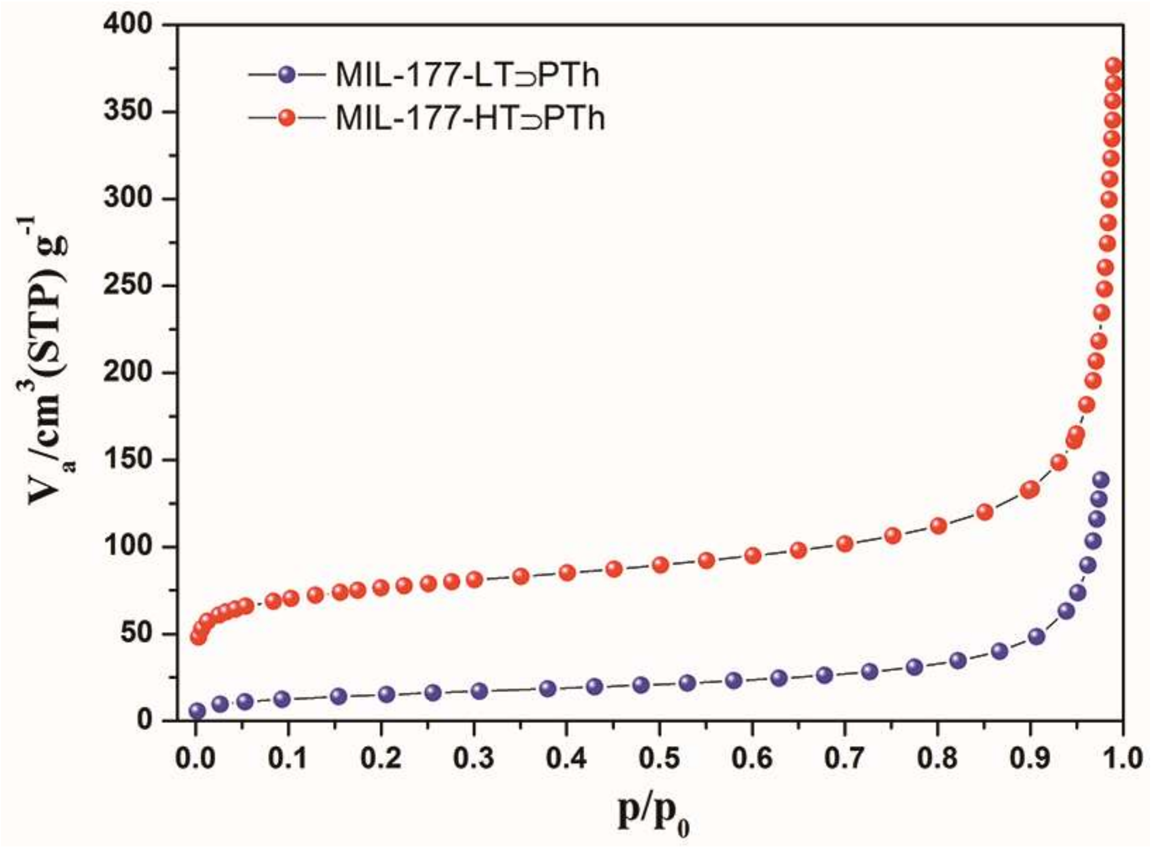

Supplementary Figure 21. Nitrogen sorption isotherms of MIL-177-LT⊃PTh (in purple) and MIL-177-HT⊃PTh (in red) composites.

The adsorption isotherms of MIL-177⊃PTh showed drastic decrease in the amount of adsorption compared with that of MIL-177, which directly indicates the presence of PTh chains within the nanochannels[15]

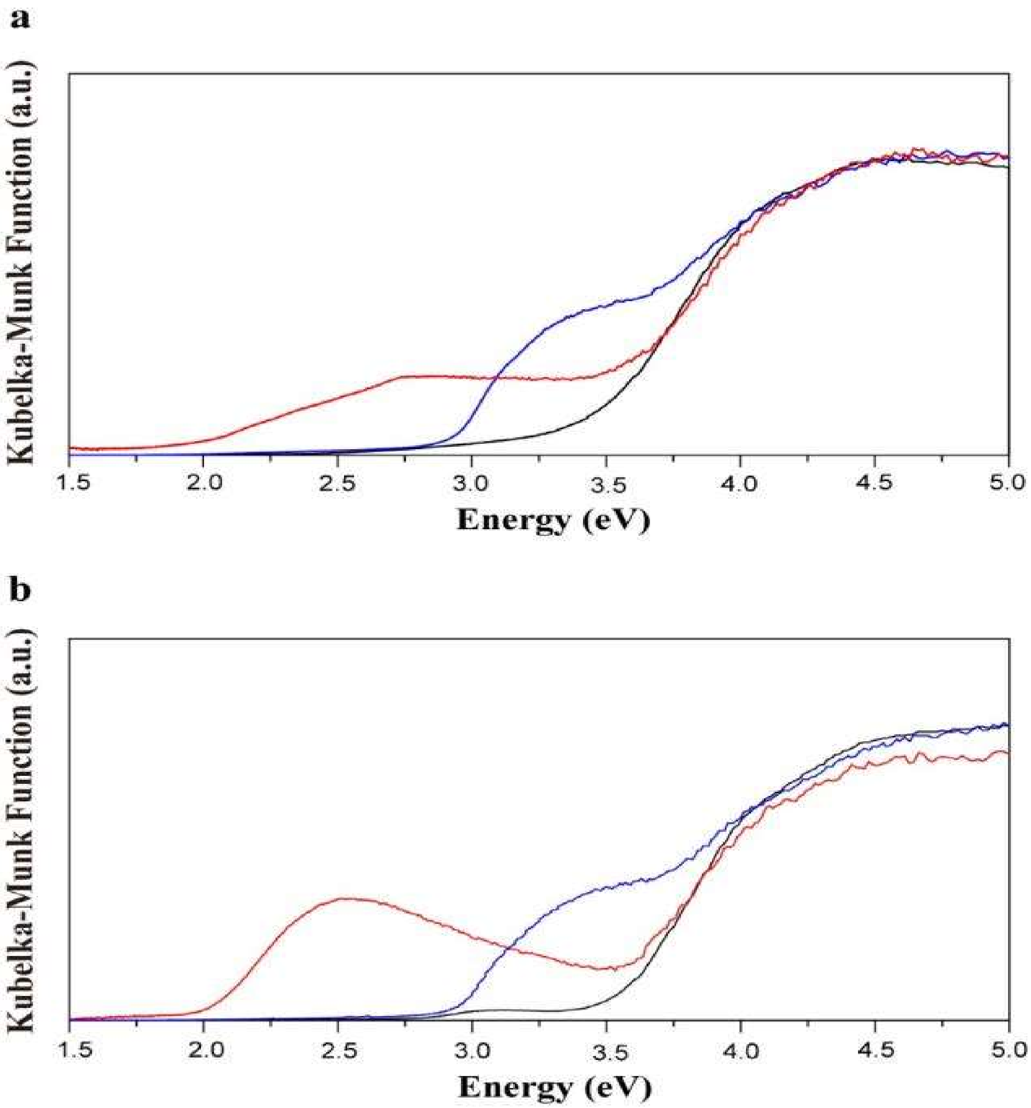

Supplementary Figure 22. Absorption spectra of **a** MIL-177-HT (black), MIL-177-HT⊃TTh (blue), MIL-177-HT⊃PTh (red) and **b** MIL-177-LT (black), MIL-177-LT⊃TTh (blue), MIL-177-LT⊃PTh (red).

The solid-state absorption spectra of MIL-177⊃PTh showed the additional absorption peak owing to the π–π* transition of neutral PTh. On the basis of the onset of the π–π* transition, the band gap of PTh is estimated to be 2.0 eV, which is comparable with reported values of PTh[16].

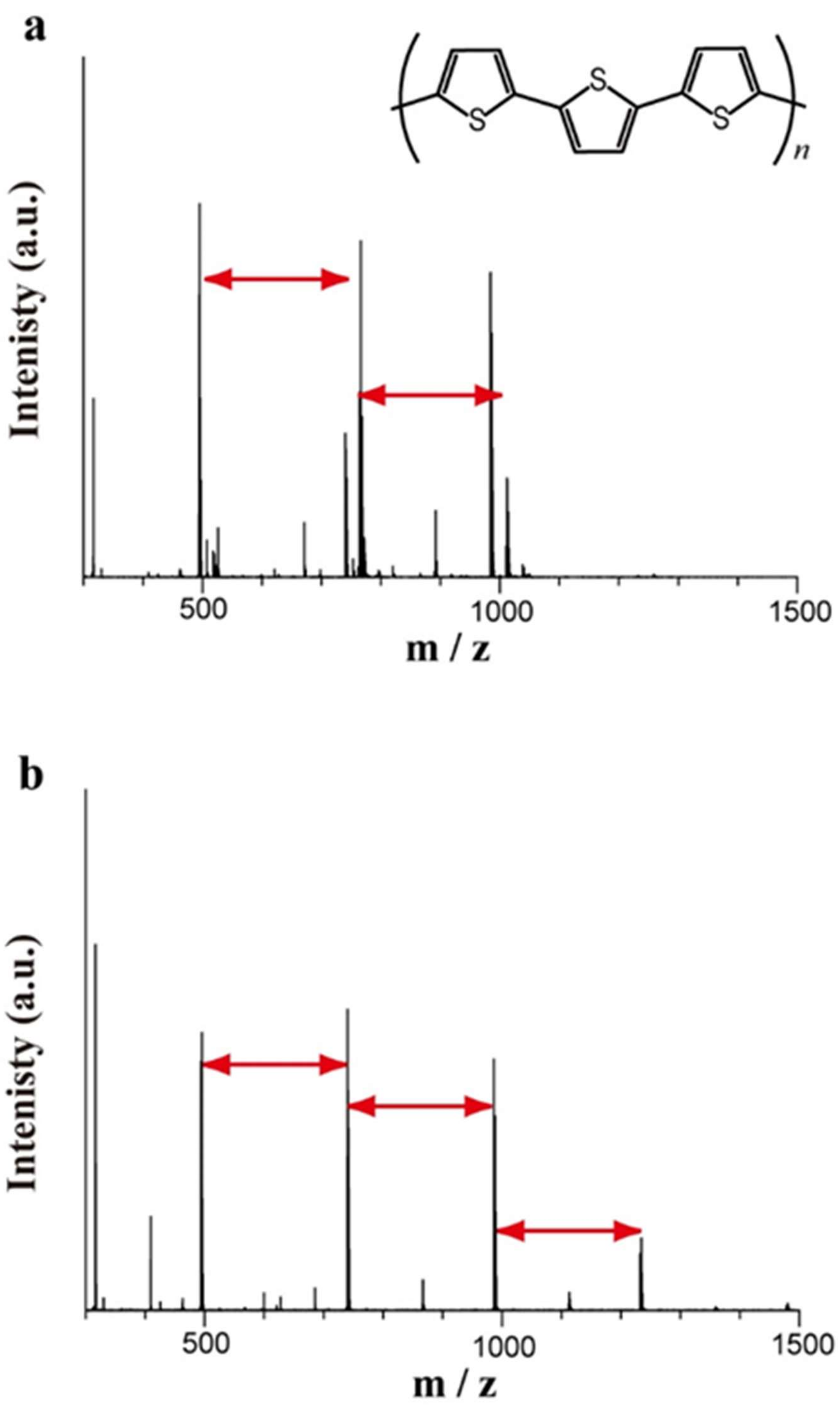

Supplementary Figure 23. MALDI-TOF mass spectra of PTh isolated from **a** MIL-177-HT and **b** MIL-177-LT.

Peaks for a number of polymers with the repeating unit of TTh (m/z = 246) could be detected.

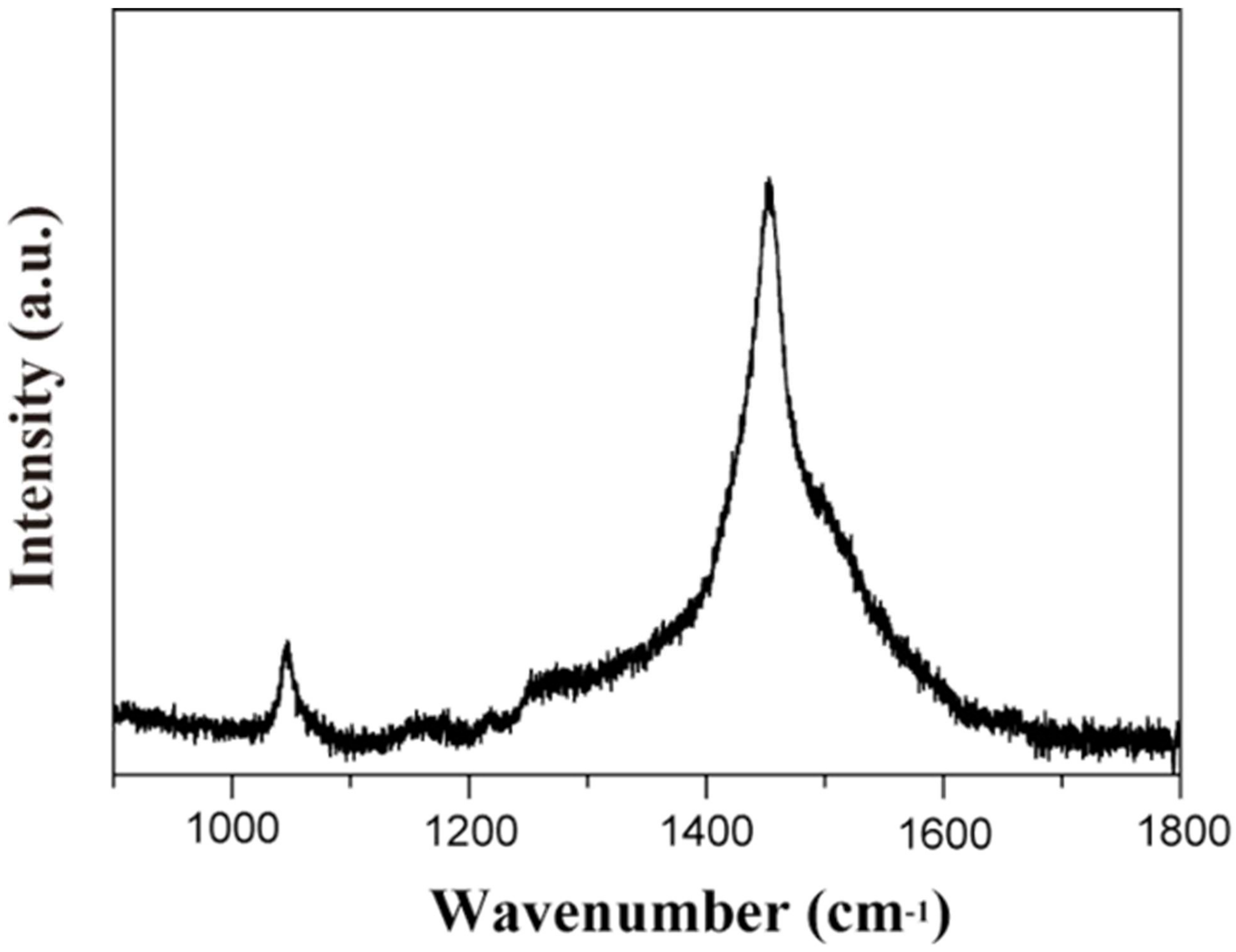

Supplementary Figure 24. Raman spectrum of PTh isolated from MIL-177-HT excited by a 532 nm laser beam.

The peaks at 1455 cm^{-1} and 1043 cm^{-1} were assigned to the C=C in-phase stretching mode and C-H bending mode of polythiophene, respectively[17].

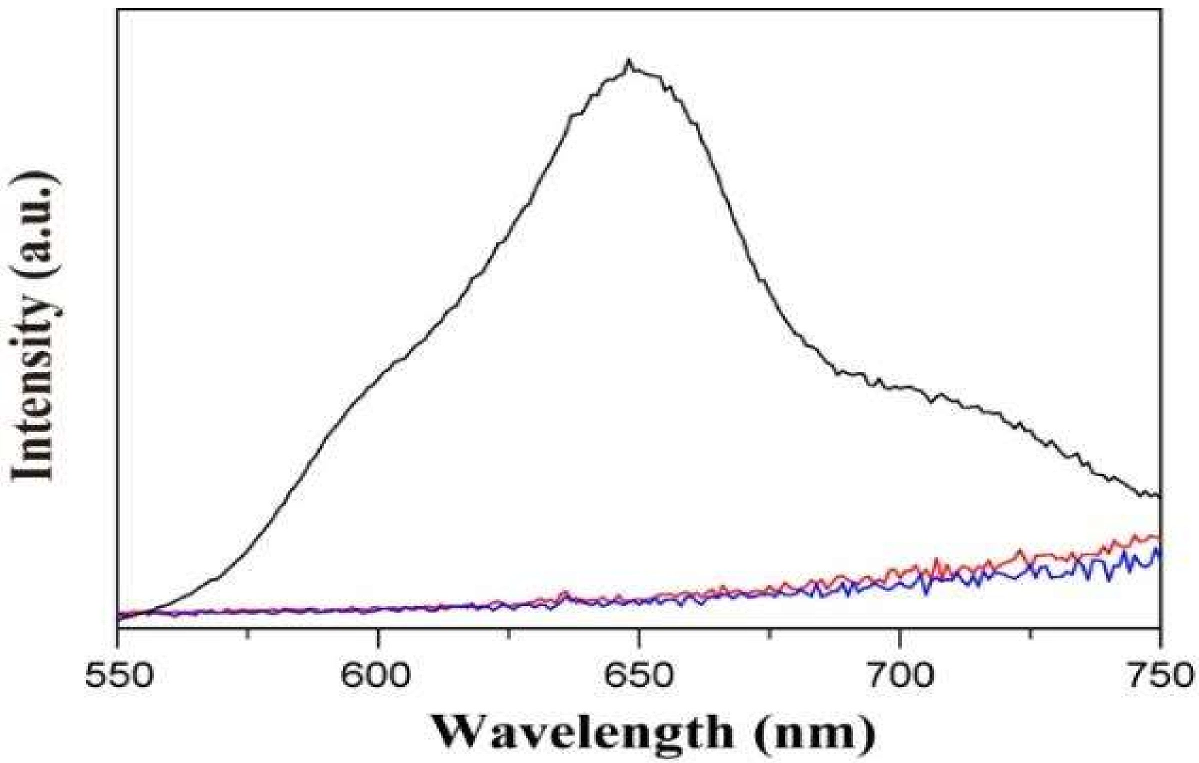

Supplementary Figure 25. Fluorescence spectra (excitation at 468 nm) for MIL-177-HT⊃PTh (red), MIL-177-LT⊃PTh (blue) and MIL-103(La)⊃PTh (black).

Polymerization of TTh was performed inside the pores of MIL-103(La) by the method similar to the preparation of MIL-177⊃PTh[18]. Taking account of the energy levels of MIL-177 estimated from UV/vis and ultraviolet photoelectron spectrum, dissociation of excitons at PTh/MIL-177 interfaces via electron injection into the conduction band of MIL-177 is thermodynamically allowed. Actually, in contrast to electro-inactive MOF, MIL-103 (La), encapsulation of PTh in the nanochannels of MIL-177 resulted in the fluorescence quenching, suggesting that charge transfer occurred from PTh to the host framework[19].

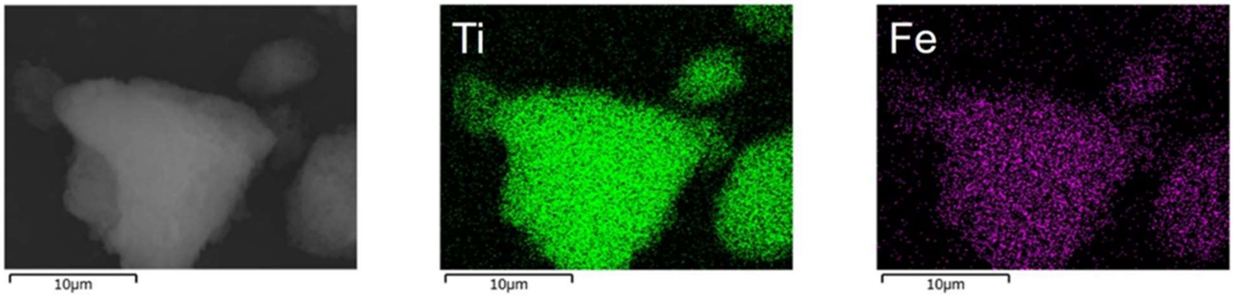

Supplementary Figure 26. SEM-EDS mapping on the first site of the Fe doped MIL-177-HT sample.

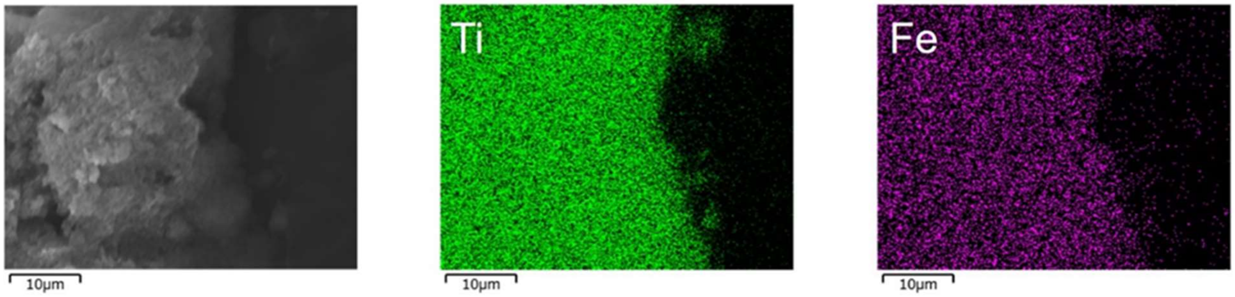

Supplementary Figure 27. SEM-EDS mapping on the second site of the Fe doped MIL-177-HT sample.

The SEM-EDS mapping result supported that the Fe dopant disperses homogeneously in the inorganic part of MIL-177-HT[20]

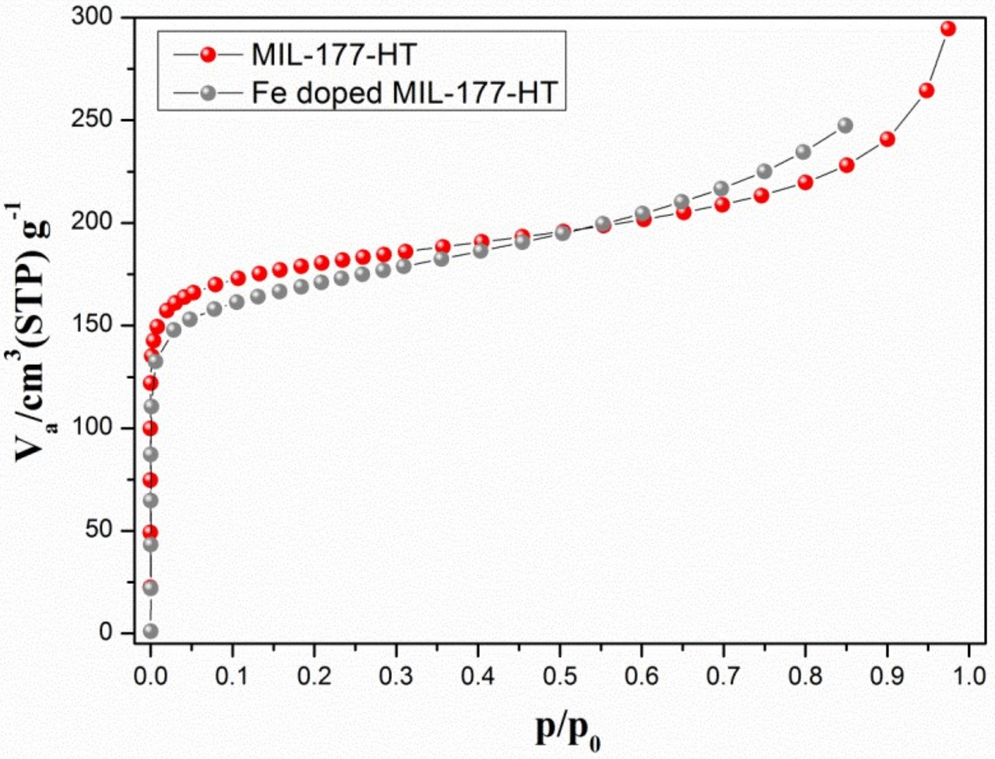

Supplementary Figure 28. Nitrogen sorption isotherms of pure MIL-177-HT and Fe doped MIL-177-HT.

Fe doped MIL-177-HT sample has a BET surface area of 636 m^2/g, a Langmuir surface area of 737 m^2/g and a total pore volume of 0.38 cm^3/g. They are all very close to the data of the pure MIL-177-HT listed in the main text when take into account the larger atomic weight of Fe compared to Ti.

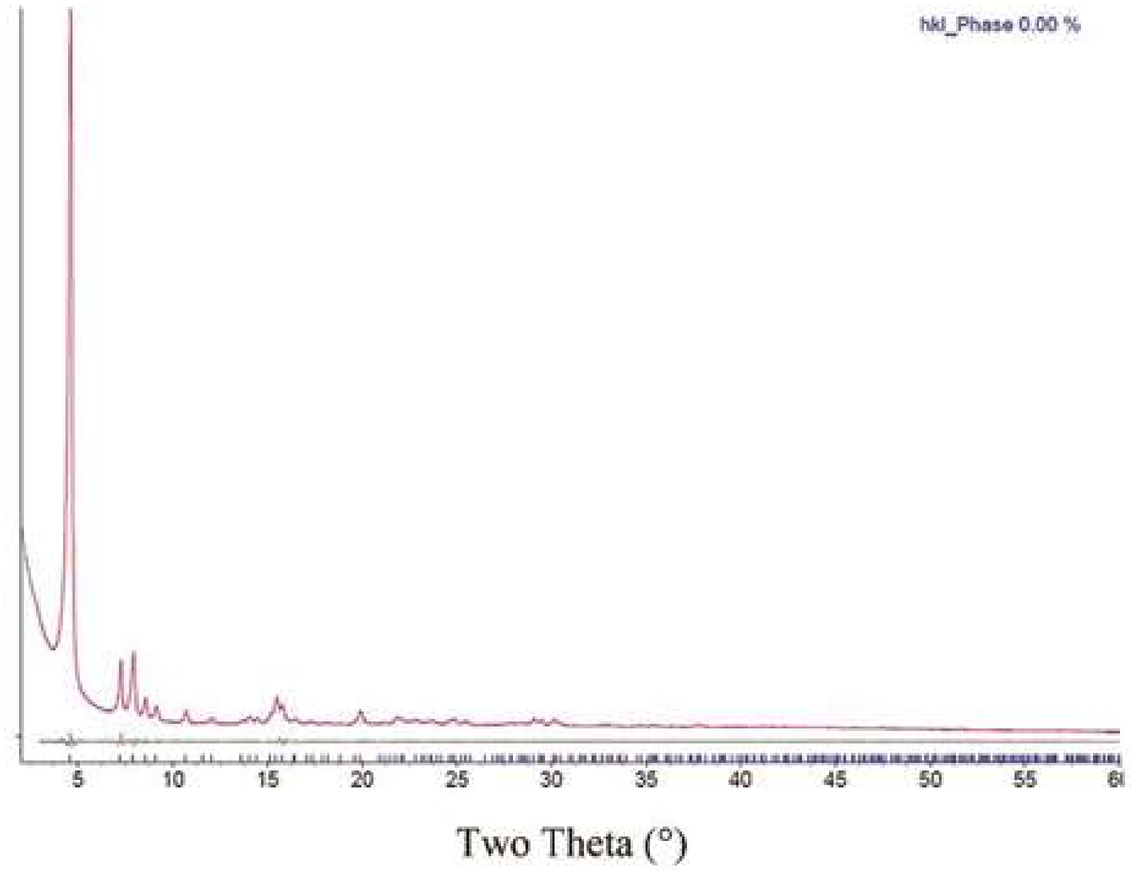

Supplementary Figure 29. Pattern matching of Fe doped MIL-177-LT sample. Space group: P6/mmm, a=22.577(2) Å, c=12.323(1) Å, Rwp=0.024.

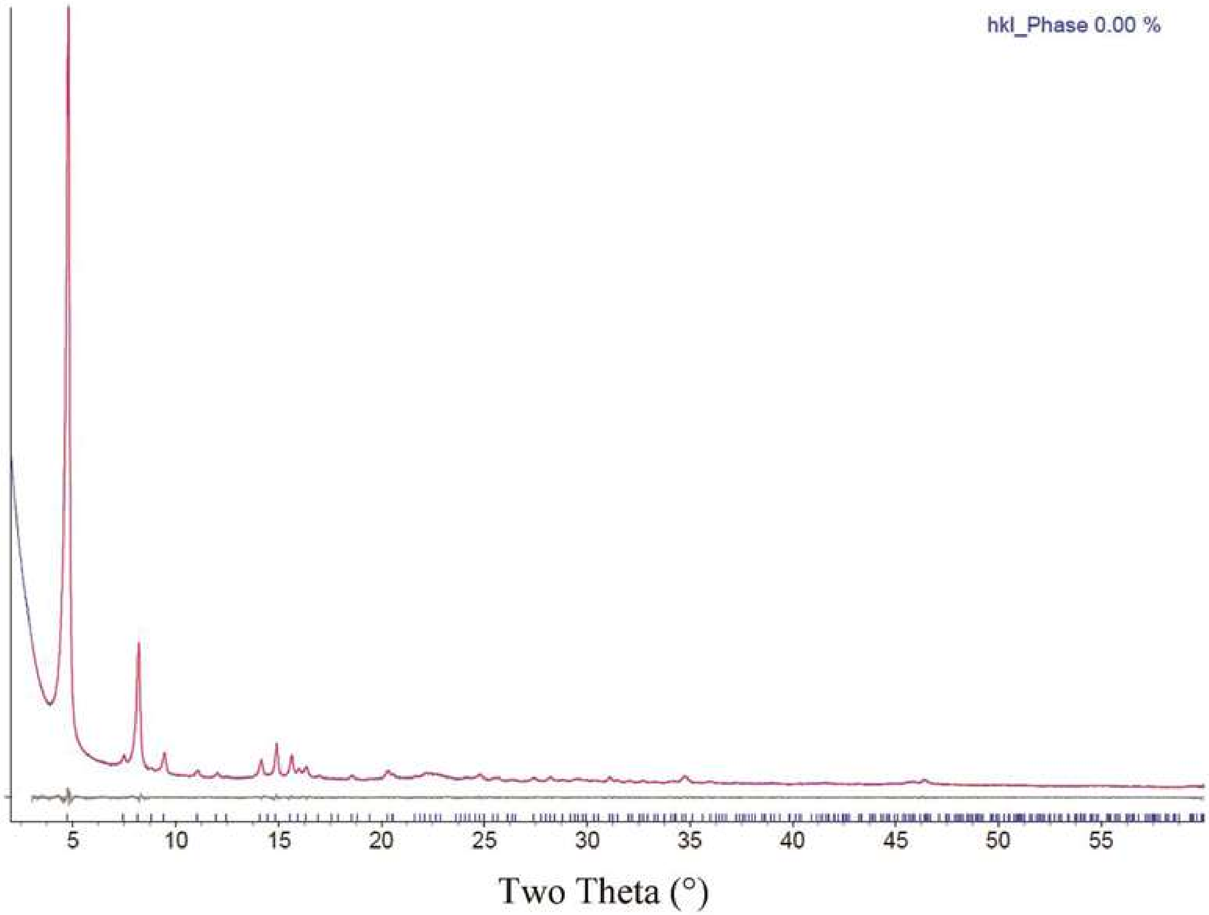

Supplementary Figure 30. Pattern matching of Fe doped MIL-177-HT sample. Space group: P6/mmm, a=21.8281(2) Å, c=11.9625(8) Å, Rwp=0.026.

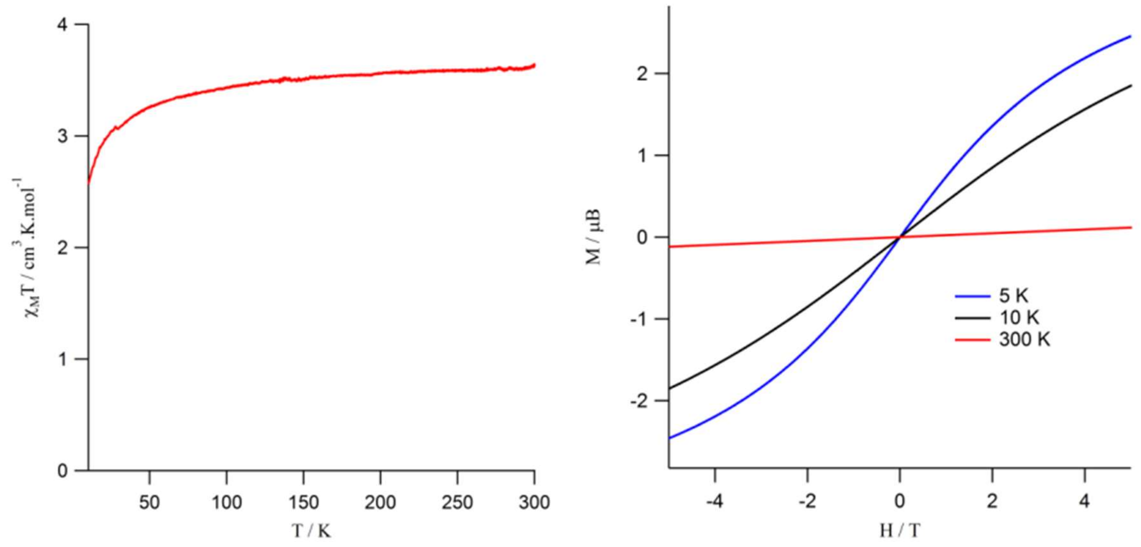

Supplementary Figure 31. (left) Evolution of the product of the molar magnetic susceptibility by the temperature $\chi_M T$ as function of temperature and (right) evolution of the magnetization as function of field at various temperatures for Fe doped MIL-177-HT.

The $\chi_M T$ product is almost constant between 50 and 300 K, indicating a paramagnetic behavior of the Fe centers. This is further confirmed by the evolution of the magnetization as function of magnetic field at various temperatures (linear at 300 K, no saturation at LT). The value of the $\chi_M T$ product of ca. 3.6 cm^3.K.mol^{-1} at 300 K is characteristic of HS Fe(III) (S=5/2). The oxidation state of Fe is also confirmed by XAS measurement at the K edge of iron. This result strongly excludes the possibility of encapsulating Fe_2O_3 in the pore of MIL-177-HT structure. Note that Fe_2O_3 should be formed during the thermal treatment for structure transformation if there is free Fe(III) species inside the porosity of MOF. Therefore, it is a solid evidence that the Fe(III) ions were doped into the inorganic unit of the MOF framework rather than staying in the pore.

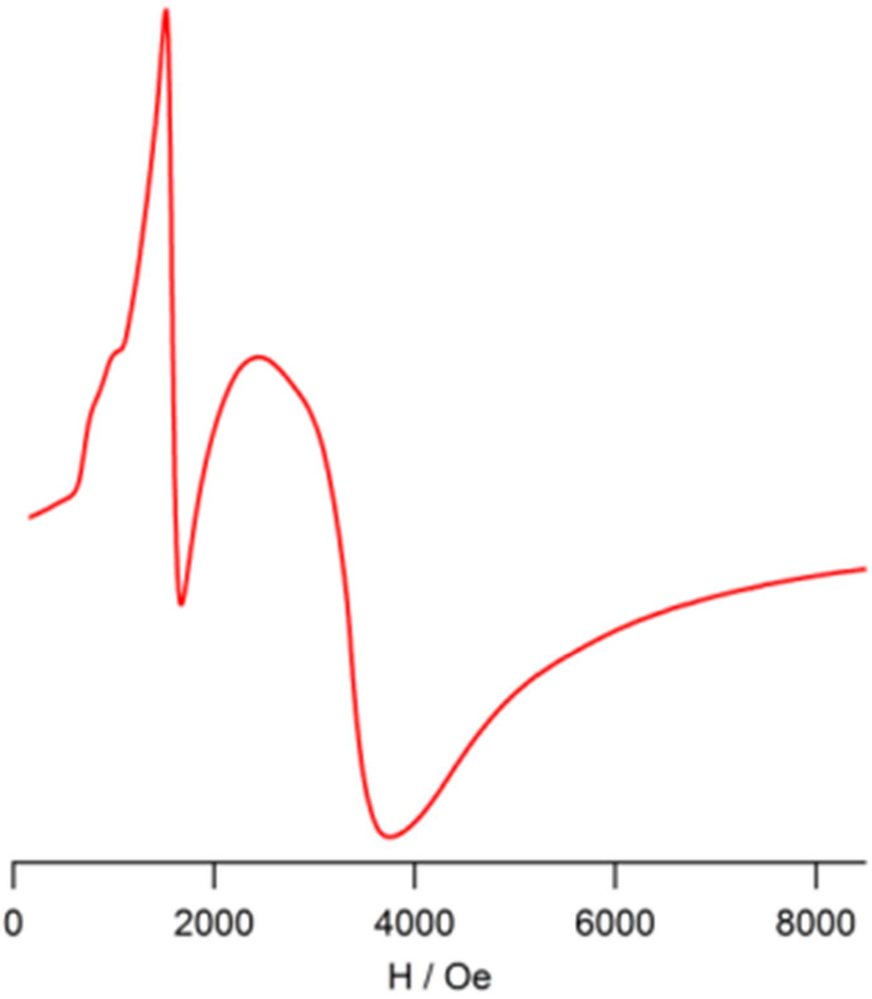

Supplementary Figure 32. EPR spectrum (X band, 10 K) of Fe doped MIL-177-HT.

The EPR spectrum of Fe doped MIL-177-HT presents two broad resonances at g = 4.23 and 2.02, which is in agreement with HS Fe(III) ions (S=5/2).

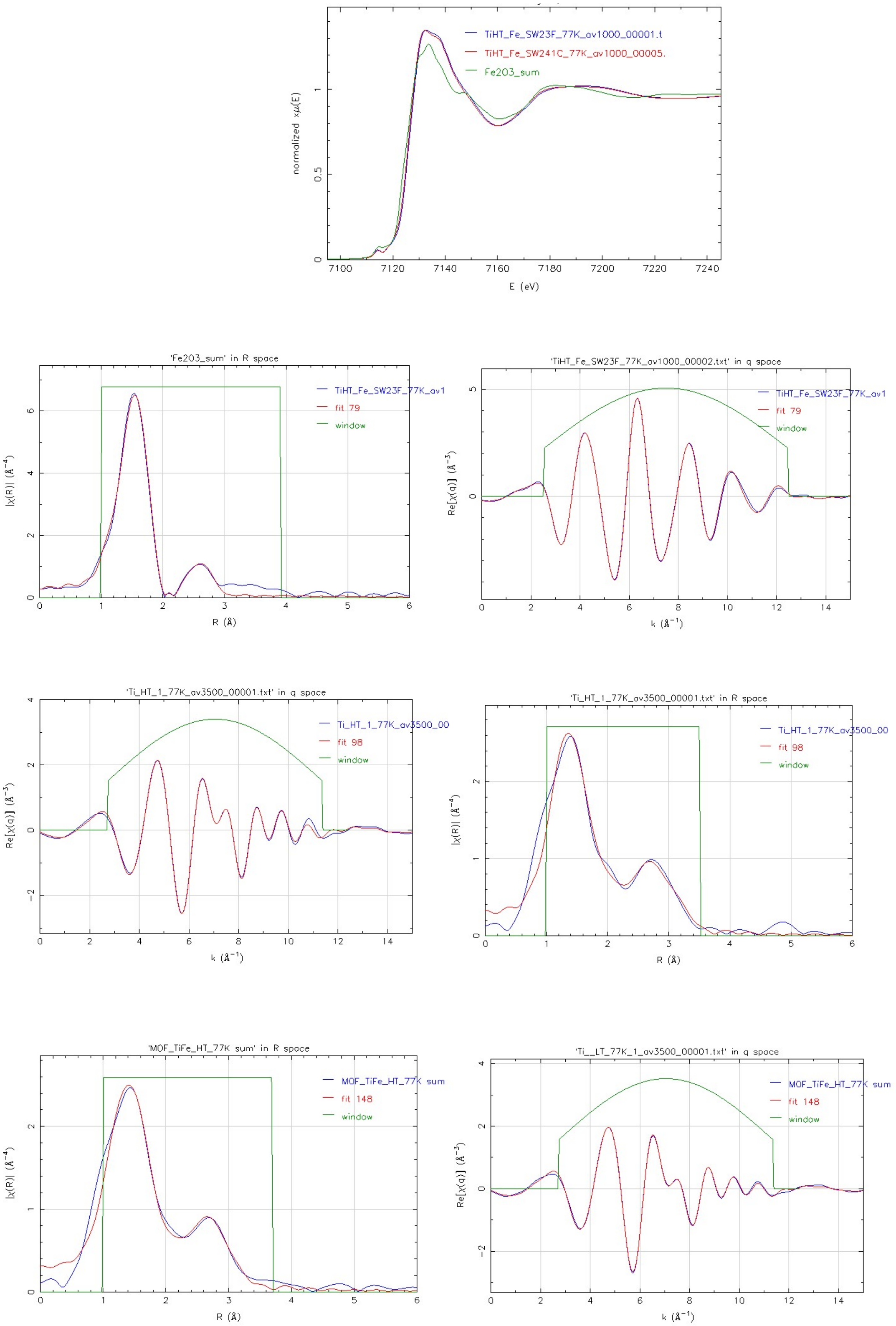

Supplementary Figure 33. EXAFS spectra and data fitting detail of the Fe doped MIL-177-HT sample.

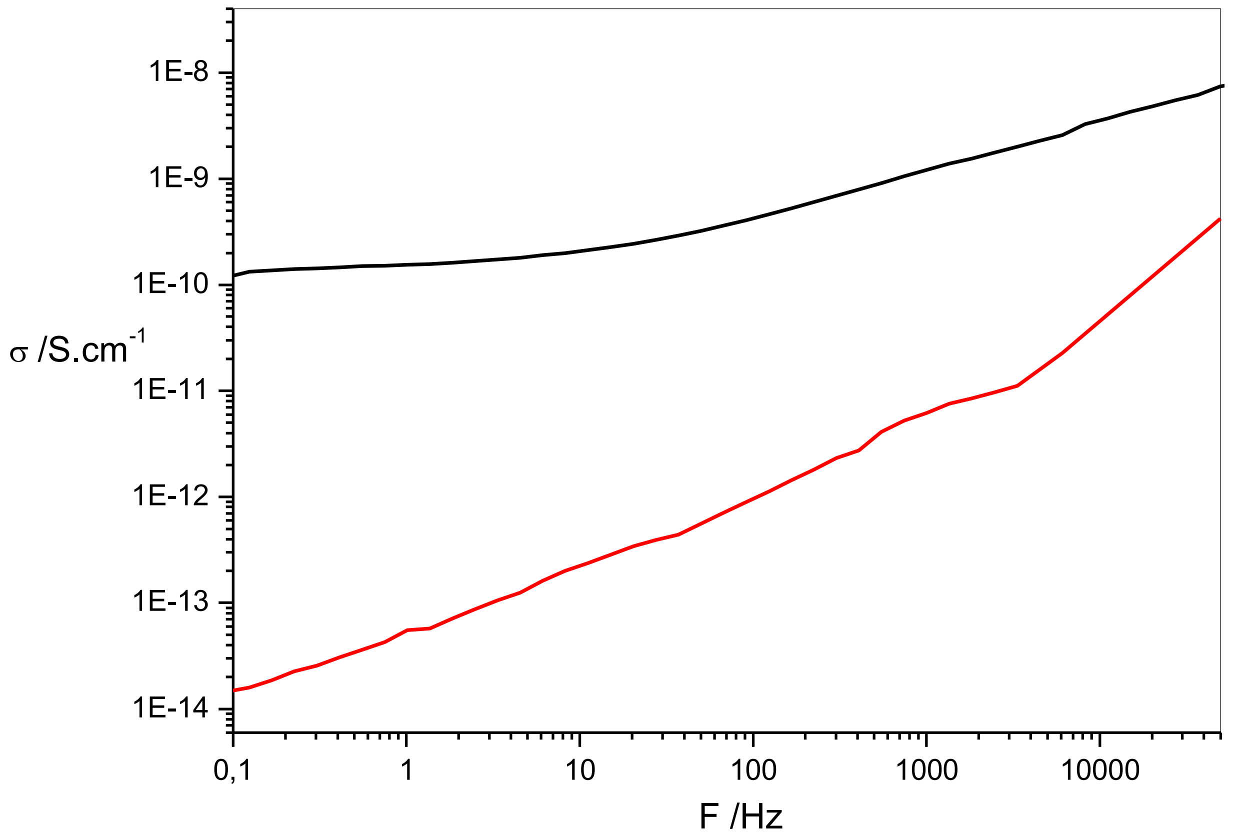

Supplementary Figure 34. Real part of the conductivity as a function of the frequency recorded at 373 K for the Fe doped (black line) and the pristine (red line) MIL-177-HT solids.

Supplementary Table 1. Crystallographic data and Rietveld refinement parameters for compounds MIL-177-LT and MIL-177-HT.

Compound	**MIL-177-LT**	**MIL-177-HT**
formula	Ti_4O_5(mdip)(formate)$_2$·2.1H_2O	Ti_4O_6(mdip)
M_r	738.95	627.76
Crystal system	Hexagonal	Hexagonal
Space group	*P*6/*mmm*	*P*6/*mmm*
a (Å)	22.5943(4)	21.8156(2)
c (Å)	12.3060(3)	11.98539(9)
V (Å^3)	5440.6(2)	4939.89(9)
M_{20}	197	374
Z	6	6
λ (Å)	1.540598	0.66973
Number of reflections	716	2115
No. of fitted structural parameters	33	34
Number of soft restraints	14	6
R_p, R_{wp}	0.023, 0.035	0.066, 0.080
R_{Bragg}, GoF	0.010, 5.56	0.013, 1.37

Supplementary Table 2. Comparison of the unit cell parameters obtained from experiment and DFT derived minimum energy geometries. The values for the angles (α= β=90 and γ=120) remained the same in experiment and theory.

**Structure phase**	**Cell parameters**	**Experiment (refined)**	**DFT (PBEsol)**
MIL-177-LT	a(= b) / Å	22.594	22.592
	c / Å	12.306	12.233
MIL-177-HT	a(=b) / Å	21.816	21.764
	c / Å	11.985	12.002

Supplementary Table 3. Comparison of experimental and calculated elemental analysis result.

MIL-177-LT	first	second	third	experimental average	calculated
**Carbon%**	28.35	28.33	28.21	28.30	30.84
**Hydrogen%**	1.81	1.65	1.72	1.73	1.92
**MIL-177-HT**	**first**	**second**	**third**	**experimental average**	**calculated**
**Carbon%**	25.98	26.02	25.79	25.93	32.48
**Hydrogen%**	1.53	1.63	1.72	1.63	1.27

Supplementary Table 4. Nitrogen sorption and EDX results of MIL-177-LT samples before and after the treatment in various acids

Chemical Treatment	S_{BET} (m^2/g)	S_{LANG} (m^2/g)	V_{pore} (cm^3/g)	EDX Result (atomic ratio)
none	730	830	0.48	-
HCl (37%)	550	622	0.39	Cl/Ti=37/63
HNO_3 (65%)	550	730	0.39	-
H_2SO_4 (98%)	700	810	0.44	S/Ti=25/75
H_3PO_4 (6M)	570	620	0.34	P/Ti=49/51
Aqua regia	790	940	0.42	Cl/Ti=19/81

The combination of nitrogen sorption and EDX results clearly show that acidic species residues trapped in the pore of MIL-177-LT after the treatment in various acids even after washing with huge amount of water.

Supplementary Table 5. SEM-EDS results obtained at five different spots on the Fe doped MIL-177-HT sample.

	**1**	**2**	**3**	**4**	**5**	**average**
**Ti (mol%)**	84.67	85.19	83.88	85.58	84.16	84.70
**Fe (mol%)**	15.33	14.81	16.12	14.42	15.84	15.30

Energy Dispersive X-ray Spectroscopy (EDS) confirmed that the Fe loading is 15.3 % (atomic ratio) while Inductively Coupled Plasma Optical Emission Spectrometry (ICP-OES) gave the Fe content of 11.01% (atomic ratio).

Supplementary Table 6. Comparison of unit cell parameters of pure MIL-177 and Fe-doped MIL-177 samples.

sample	*a*=*b* (Å)	*c* (Å)	*V* ($Å^3$)
**MIL-177-LT**	22.5397(8)	12.2832(5)	5404.3(4)
**Fe-doped MIL-177-LT**	22.577(2)	12.323(1)	5440(1)
**MIL-177-HT**	21.7412(7)	11.9376(3)	4886.7(3)
**Fe-doped MIL-177-HT**	21.8281(2)	11.9625(8)	4936.1(8)

PXRD diagrams for pattern matching of pure MIL-177 and Fe-doped MIL-177 samples were all collected on a high-throughput Bruker-D8 Advance diffractometer in transmission mode. As the crystal ionic radii for Fe(III) (78.5 pm) is just slightly larger than that of Ti(IV) (74.5 pm), thus the unit cell parameters of the Fe-doped MIL-177 samples are slightly larger than those of the pure MIL-177 samples.

Supplementary Table 7. Result summary of fits at the Fe K edge.

Fe K edge	N	R (Å)	σ^2(Å^2) 10^{-3}	Statistical fitting parameters
Fe-doped MIL-177-HT sample 1	5.7 ± 0.3 O<br>1.8 ± 0.7 Ti	1.99 ± 0.003<br>3.08 ± 0.014	7.9 ± 0.6<br>13.1 ± 0.5	χ^2 = 426.7<br>χ_υ^2 = 172.6<br>R-factor = 0.000439399
Fe-doped MIL-177-HT sample 2	5.5 ± 0.2 O<br>1.5 ± 0.5 Ti	1.99 ± 0.003<br>3.06 ± 0.012	7.6 ± 0.5<br>11.7 ± 3.6	χ^2 = 328.5<br>χ_υ^2 = 118.1<br>R-factor = 0.000443482

Supplementary Table 8. Result summary of fits at the Ti K edge.

Ti K edge	N	R (Å)	σ^2(Å^2) 10^{-3}	Statistical fitting parameters
Pure MIL-177-HT	3 O<br>2 O<br>2 Ti<br>2 C<br>2 Ti<br>2 Ti	1.87 ± 0.005<br>2.04 ± 0.007<br>3.06 ± 0.005<br>3.17 ± 0.014<br>3.40 ± 0.010<br>3.68 ± 0.016	10.4 ± 1.1<br>8.9 ± 1.5<br>10.4 ± 1.5<br>0.1 ±2.5<br>10.4<br>10.4	χ^2 = 12522<br>χ_υ^2 = 5008<br>R-factor = 0.000559299
Fe-doped MIL-177-HT	3 O<br>2 O<br>2 Ti<br>2 C<br>2 Ti<br>2 Ti<br>0.26 Fe	1.85 ± 0.012<br>2.00 ± 0.008<br>3.05 ± 0.010<br>3.15 ± 0.020<br>3.37 ± 0.016<br>3.64 ± 0.026<br>3.08	10.6 ± 2.2<br>11.8 ± 1.9<br>11.1 ± 0.6<br>0.1<br>11.1<br>11.1<br>13.0	χ^2 = 30928<br>χ_υ^2 = 9400<br>R-factor = 0.000520832

Supplementary Note 1, Structure determination of MIL-177-LT and MIL-177-HT

The powder diffraction data of MIL-177-LT were measured at room temperature on a Bruker D8 Advance diffractometer with a Debye-Scherrer geometry, in the 2θ range of 2-80 °. The D8 system is equipped with a Ge(111) monochromator producing Cu $K\alpha_1$ radiation (λ = 1.540598 Å) and a LynxEye detector.

High-resolution X-ray powder diffraction data of MIL-177-HT were collected on the CRISTAL beamline at Soleil Synchrotron (Gif-sur-Yvette, France). A monochromatic beam was extracted from the U20 undulator beam by means of a Si(111) double monochromator. Its wavelength of 0.66973 Å was refined from a LaB_6 (NIST Standard Reference Material 660a) powder diagram recorded prior to the experiment. High angular resolution was obtained with, in the diffracted beam, a 21 perfect crystal Si(111) multi-analyser similar to the one employed on beamline ID31 at ESRF[21]. The sample was loaded in a 0.7 mm capillary (Borokapillaren, GLAS, Schönwalde, Germany) mounted on a spinner rotating at about 5 Hz to improve the particles' statistics. Diffraction data were collected for less than 2 h in continuous scanning mode and the diffractogram was obtained from the precise superposition and addition of the 21 channels data.

Extractions from the peak positions, pattern indexing, whole powder pattern decomposition, direct space strategy used to complete the structural models as well as difference Fourier calculations and Rietveld refinements were carried out with the TOPAS program[22]. In both cases, the LSI-indexing method converged unambiguously to a hexagonal unit cell without systematic extinctions. The structural determination of the MIL-177-LT was initialized with the EXPO package[23], using EXTRA for extracting integrated intensities and SIR97 for direct-methods structure solutions. This allowed in the *P*622 space group to localize two independent Ti cations with some oxygen atoms of their environment as well as the central carbon atom of the mdip moiety. The direct space

strategy was then used to complete the structural model and half of a linker has been considered as rigid body, added to the partial structural model, and allowed to rotate around its central carbon. Guest water molecules and carbon atoms of formates where then located by difference Fourier maps. Taking into account the atomic coordinates of the framework solely, Platon[24] was used to check for a higher symmetry and it was deduced that the structure was centric (*P*6/*mmm* space group). At its final stage (Table S1 and Figure S1), the Rietveld refinement of MIL-177-LT involved the following structural parameters: 21 atomic coordinates, 1 translation and 1 rotation parameters for the mdip linker, 3 distances and 1 rotation angle for the rigid body, 4 occupancy factors of the water molecules, 1 thermal global factor and 1 scale factor.

Regarding similarities between unit cells of LT and HT phases, the framework of the MIL-177-LT (after removing formate groups) was firstly used as the starting point of the Rietveld refinement of the MIL-177-HT but this did not converge to a reliable solution. The same strategy as used for the MIL-177-LT was then applied to solve the structure of the MIL-177-HT, but even with synchrotron data, accurate atomic coordinates were difficult to obtain. An unrefined structural model was then fully optimized at the density functional theory (DFT) level (see below for details). At its final stage, the Rietveld refinement MIL-177-HT (Table S1), involved the following structural parameters: 30 atomic coordinates, 3 thermal factors and 1 scale factor.

Supplementary Note 2, DFT calculation for structure determination

The unrefined crystallographic positions of the atoms of MIL-177-LT and MIL-177-HT were used to build models of the corresponding structures, which were then fully optimized at the density functional theory (DFT) level, allowing both the atomic positions and the unit cell parameters to vary. These calculations were performed using the Quickstep DFT[25] module of the CP2K code[26].

All atoms were treated using a combined Gaussian and planewave basis set. The PBEsol[27] functional was used along with a combined Gaussian basis set and plane wave pseudopotential strategy as implemented in the code. A triple-ζ Gaussian-type basis set (TZVP-MOLOPT basis set provided with the code) was considered for all atoms, except for the Ti metal centers, where double-ζ functions were employed (DZVP-MOLOPT)[28]. The pseudopotentials used for all of the atoms were those derived by Goedecker, Teter, and Hutter[29]. These calculations included the semi-empirical dispersion corrections as implemented in the DFT-D3 method, derived by Grimme[30]. The resulting models were used as a starting point for further Rietveld refinement. This leads to satisfied structure models of MIL-177 as evidenced by the Rietveld plots (Figure S1 and S2).

Supplementary Note 3, Elemental analysis

The first and second batches of data were obtained from the test carried out by Service de Microanalyse, I.C.S.N.-CNRS, 91198, Gif sur Yvette Cedex. The third batch of data were collected by Institut Des Sciences Analytiques-CNRS.

The major differences of the carbon content for MIL-177-LT between the one calculated from the crystal structure and experimental finding possibly could be ascribed to the presence of defects in the MOF structure, which is quite normal for MOFs obtained in the reaction conditions containing large amount of modulator solvents. In the case of MIL-177-HT, the thermal treatment which causes the irreversible phase transformation likely results in even more structural defects in the MOF framework, corresponding to the even larger difference of the calculated carbon content compared with the experimental one.

There are two kinds of structural defects observed in the MOFs structures according to the chemical components, the missing linker or missing inorganic building unit. The SBU defect generally will lead to higher linker percentage compared with the calculated data on the non-defect sample, which results in higher elemental analysis result on carbon. Thus it is not the case for the MIL-177 compounds. Instead, the linker defect is taken into consideration here. For the LT compound ($Ti_4O_5(mdip)(formate)_2 \cdot 2.1H_2O$), when x mdip molecule is missing, there will be 4x formate groups to take over the coordination sites left from the missing mdip carboxylate groups. In this case the carbon percentage could be calculated as below:

$$[(1-x)*17*12+(2+4x)*12]/[739-340x+180x] = 0.283$$

This equation gives the x value of 0.17, which means 17% of the mdip linker in the LT structure is missing, giving rise to a formula for the LT with defect as $Ti_4O_5(mdip)_{0.83}(formate)_{2.68} \cdot 2.1H_2O$. It corresponds to the TGA curve of LT experimental data very well if the instrument system error is taken into consideration (organic part: 61% Cal vs 59% Found).

Similarly, for HT phase, once there is one mdip linker molecule missing, there will be 4 -OH and 4 water to take over the coordination sites while keeping the charge balance, resulting in the following equation for the carbon percentage in the defect containing HT sample:

$$[(1-x)*17*12]/[628-340x+140x] = 0.2593$$

The x value solved from the equation is 0.27, suggesting 27% mdip linker are missing in the HT structure. Therefore the defect HT sample has a formula of $Ti_4O_6(mdip)_{0.73}(OH)_{1.08}(H_2O)_{1.08}$. This formula matches with the TGA result well as the similar case for the defect LT MOF (organic part: 50% Cal vs 49% Found).

Supplementary Note 4, Chemical stability test result and discussion

NH_2-MIL-125, generally considered as a benchmark in the field of Ti-MOFs, was used as the reference to study the chemical stability of both MIL-177-LT and MIL-177-HT samples. It was found that the crystallinity of NH_2-MIL-125 sample decreased slowly in water at room temperature and eventually its PXRD pattern became flat when we checked it at the 22nd day. It showed very high sensitivity towards hot water. When 50 mg of the sample was heated in 20 mL of water at 60 °C only for 12 hours, the PXRD of the resulting solid did not show any peaks corresponding to the original structure. When acidic conditions were applied to the sample of NH_2-MIL-125 at room temperature, it was found that the MOF solid could completely dissolved in HCl (1M), HNO_3 (1M) and H_2SO_4 (1 M) forming clear light yellow solutions after soaking for two hours. H_3PO_4 with a concentration of 0.2 M could completely damage the MOF structure within two hours. In addition, NH_2-MIL-125 displayed very limited resistance towards basic condition. It became almost amorphous according to its PXRD pattern which was collected after 24 hours soaking in NaOH/$NaHCO_3$ buffer (pH=10) at room temperature.

In sharp contrast to NH_2-MIL-125, the MIL-177-LT sample has an excellent stability in water and extreme acidic conditions at room temperature. The crystallinity of the MOF structure maintained very well under the above mentioned conditions for a long time. The compound also shows a very good hydrothermal stability and tolerance in basic condition, without notable change in its PXRD patterns compared to the as-made sample.

MIL-177-HT exhibits weaker chemical stability than that of the MIL-177-LT compound in general. For example, the MIL-177-HT solid was not able to survive in concentrated acid solutions. But it showed good tolerance under diluted acid conditions (note that these diluted acid conditions are still very acidic). Regarding the water stability, MIL-177-HT sample is as good as the MIL-177-

LT one with an evidence of an unchanged PXRD pattern of sample checked after 35 days soaking in water at room temperature. Hot water (such as 60 °C, 70 °C and 80 °C) did not show too much destructive power to its crystallinity in 48 hours. But we found that it was completely destroyed in boiling water after 24 hours. Its tolerance in basic condition is comparable with that of NH_2-MIL-125, but clearly much worse than that of the MIL-177-LT form structure. Therefore the overall chemical stability of MIL-177-HT still outperforms notably the NH_2-MIL-125 sample, but clearly worse than that of MIL-177-LT which probably due to considerable amount of structural defects generated during the thermal treatment for phase transformation which involves complicated bond cleavage, connection rearrangement and bond reformation.

Supplementary Note 5, Fe and Ti K edges EXAFS characterization

A batch of Fe doped MIL-177-HT sample with 13% Fe content (atomic ratio in comparison with Ti in the MOF structure, determined by EDS) was used for the Fe and Ti K edges EXAFS characterization.

The EXAFS spectra were Fourier transformed using a Kaiser-Bessel window with dk = 2. The k-range used for fitting (Δk) was 6.7 $Å^{-1}$ at the Ti K edge and 8 $Å^{-1}$ at the Fe K edge whereas the R-range (ΔR) was 2.5 Å at the Ti K edge and 2.1 Å at the Fe K edge. Anatase TiO_2 and crystalline α-Fe_2O_3 samples were first used as references for the EXAFS data analysis using the Athena and Artemis graphical interface programs (ref). The amplitude reduction factor S_0^2 for Ti K edge and Fe K edge analysis was determined at 0.98 and 0.83, respectively and kept fixed to these values for fitting pure MIL-177-HT and Fe-doped MIL-177-HT samples. The E_0 parameter was allowed to vary during fitting at the Fe K edge and variation is 7127.2 ± 0.2 eV whereas this parameter was

kept fixed to the value determined on the anatase TiO_2 at 4981.9 eV. Least square fits of the structural parameters R (average atomic distance from the absorbing atom), N (coordination number) and σ^2 (Debye-Waller factor) were performed using simultaneous k-weighting of 1, 2 and 3. The coordination numbers for the different shells of neighbours around Ti were kept fixed to the crystallographic values extracted from the cif file obtained for the pure MIL-177-HT sample whereas the coordination numbers for the two shells of neigbhours around Fe were varied. For the fit of the Fe-doped MIL-177-HT sample, an additional contribution of Ti-Fe has been added to the crystallographic distance determined from the fitting of the second nearest coordination shell around Fe (Fe K edge) and identified as a Titanium contribution. The number of variables used in the fit, Nvar, was always lower to the independent number of parameters determined according to the formula Nidp = $(2\ \Delta k\ \Delta R/\pi) + 2$[31]. The goodness of fit is given by the minimum value of the statistical χ^2 metric parameter and of the reduced quality factor χ_{υ}^2 defined as the ratio of χ^2 over υ, where υ = Nidp – Nvar.

The rising edge measured at the Fe K edge for the Fe-doped MIL-177-HT sample is at the same position in energy than the one measured for the crystalline α-Fe_2O_3 sample, strongly suggesting Fe(III) as valence state for iron in the doped MOF-Ti network. Table S6 and S7 report the results of the fits at the Fe and Ti K edges. Additionally the simulation of the EXAFS spectra (Table S6) recorded for the Fe-doped MIL-177-HT sample indicates a first coordination shell of 6 oxygen atoms and as second nearest neighbours ≈ 2 titanium atoms at 3.08 Å. This second contribution is fully consistent with the substitution of titanium by iron. As a matter of fact, a satisfactorily simulation of the Ti K edge EXAFS spectrum of the Fe-doped MIL-177-HT can be achieved by imposing the presence of an iron contribution with structural parameters (R and σ) fully constrained by the results of the fit at the iron K edge (see Table S7) and coordination number in

agreement with the complete substitution of 13 at% of titanium by iron. The reduced quality factor χ_{υ}^2 with the constrained Iron contribution is found at 9400 against 14740 without this contribution pointing out that the addition of this constrained contribution is necessary to fully described the local order around Ti in the Fe-doped MIL-177-HT sample.

Supplementary Note 6, Conductivity measurements

Impedance measurements were performed on a broadband dielectric spectrometer, Novocontrol alpha analyzer, over a frequency range from 1 Hz to 1 MHz with an applied ac voltage of 1 V. The temperature of the sample was controlled by the Quatro Novocontrol system. Measurements were collected from 373 K to 298 K on the anhydrous solid, obtained by *in situ* heating at 373 K for 2 hours. The powder sample was placed in a home-made cell, i.e. about 75 mg of the solid were inserted between two gold electrodes in a parallel plate capacitor configuration with an annular Teflon spacer for insulation, allowing the use of the two-probe method to perform the electrical measurements.

Typically, the real part of the ac conductivity, $\sigma_{ac}(\omega, T)$, results from the combination of three contributions: $\sigma_{ac}(\omega, T) = \sigma_{MWS}(\omega, T) + \sigma_{dc}(T) + \sigma'(\omega, T)$. The polarization component $\sigma'(\omega, T)$, corresponding to the increasing part of the signal observed at high frequency, arises from a local rearrangement of charges or dipoles causing dipolar reorientation. The *dc* conductivity plateau $\sigma_{dc}(T)$, resulting from the long-range redistribution of charges dominates the intermediate frequency region. In case of highly conductive materials, the Maxwell Wagner Sillars contribution $\sigma_{MWS}(\omega, T)$ due to the charge accumulation to the sample/electrode interface is observed at low frequency. In insulators, $\sigma_{MWS}(\omega, T) \approx 0$ and σ_{dc} (T) ≈ 0 and only the polarization conductivity is detectable.

Supplementary Methods

Materials 5,5'-methylenediisophthalic acid (H_4mdip) was synthesized according to the reported procedure in the literature[32]. Ti(*i*PrO)$_4$ (Alfa Aesar, 97%), formic acid (Acros, 98+%), sulfuric acid (Sigma-Aldrich, 98%), phosphoric acid (Sigma-Aldrich, 85 wt% solution in water), hydrochloric acid (Prolabo, 37%), nitric acid (Fisher, 65%), anhydrous $FeCl_3$ (Alfa, 98%), terthiophene (TCI, 98%), iodine (Wako, 99+%) were used as received from commercial suppliers without further purification.

Measurements The Powder X-ray diffraction (PXRD) data for structure solution of MIL-177-LT were collected with a Bruker D8 Advance diffractometer with a Debye-Scherrer geometry, whereas synchrotron data on the MIL-177-HT compound were recorded at Soleil Cristal Beamline (λ = 0.66973 Å) (see structure determination part for more details). Routine PXRD data for general characterization were recorded on a high-throughput Bruker D8 Advance diffractometer working on transmission mode and equipped with a focusing Göbel mirror producing CuKα radiation (λ = 1.5418 Å) and a LynxEye detector. PXRD diagrams for all the samples in MIL-177⊃PTh composites part were collected on a Rigaku SmartLab Diffractometer, using a Cu anode (λ = 1.5418 Å). X-ray thermodiffraction was performed using a θ-θ Bruker-D8 Advance diffractometer equipped with a HTK-1200N (Anton Parr) high-temperature chamber and a LYNXEYE XE detector (Cu radiation). PXRD patterns were collected every 25 °C from room temperature to 500 °C, with two hours scan for each temperature. Infrared spectra were measured with a Nicolet 6700 FTIR thermoscientific spectrometer between 400 and 4000 cm^{-1}. Thermogravimetric analyses were carried out with a Mettler Toledo TGA/DSC 1, STAR System apparatus under an O_2 flow of 50 mL/min, at a heating rate of 3 °C/min to 600 °C. Solid-state NMR spectra were recorded with an Advance Bruker 500 NMR spectrometer (B0 = 11.7 T, corresponding to Larmor

frequencies of 500.1, and 125.7 MHz for ^{1}H and ^{13}C respectively). The samples were packed either in 4 or 2.5 mm outer diameter rotors, with respective MAS rates of 10 and 30 kHz. Scanning Electron Microscope (SEM) images and EDS data of MIL-177 MOFs were taken with a JEOL JSM-7001F microscope using gold coated samples equipped with an energy-dispersive X-ray (EDX) spectrometer with a X-Max SDD (Silicon Drift Detector) by Oxford. SEM-EDS measurements on Fe doped MIL-177-HT sample, MIL-177⊃PTh composites and isolated PTh were conducted using a HORIBA EMAXEvolution EX-370 attached to a HITACHI S-3000N operated at an accelerating voltage of 30 kV. Samples were placed on a conducting carbon tape attached by SEM grid, and then coated with platinum. Ultraviolet-visible (UV-vis) spectra were recorded on a JASCO V-670 spectrometer with samples diluted in $BaSO_4$ at room temperature. Nitrogen sorption measurements were performed with a BEL Japan Belsorp Mini apparatus at 77 K after the sample being fully activated (BEL Japan, BELSORP Prep). Ultraviolet photoelectron spectroscopy was carried out on PCR-102 (Sumitomo Heavy Industries Advanced Machinery Co. Ltd. Monochlomated UV excitation light was irradiated under vacuum (< 10^{-5} Pa), and ejected electrons were corrected and measured by a Keithley 6430 sub-femto source meter with an ITO reference substrate. Flash-Photolysis Time Resolved Microwave Conductivity (FP-TRMC) measurements were performed under illumination of the third (355 nm) and forth (266 nm) harmonic generation from a nano-second INDI-HG Nd:YAG laser (Spectra-Physics Inc.) operated at 10 Hz. The solid samples were bound on quartz substrates with poly(methylmethacrylate) matrices, and mounted in Ar, O_2, or Air-filled microwave cavity. The frequency and power of the probing microwave injected into the cavity was tuned from 9.05 -9.09 GHz and 3 mW, respectively. Matrix-assisted laser desorption/ionization time-of-flight mass spectrometry (MALDI-TOF MS) spectra were recorded on an ultraflex instrument (Bruker Daltonics) using terthiophene as the

matrix. Raman spectra were collected on a HORIBA LabRAM HR-Evolution spectrometer, using a laser source (wavelength: 532 nm) for excitation. Fluorescence spectra were measured on Fluorolog-3 (HORIBA Jobin Yvon Inc.) with double-grating monochromators, 450-W xenon lamp, and R928 photomultiplier tube run in photon-counting mode. Impedance measurements were performed on a Broadband Dielectric Spectrometer, Novocontrol alpha analyzer over a frequency range from 1 Hz to 1 MHz with an applied ac voltage of 1 V. The temperature of the sample was controlled by the Quatro Novocontrol system. Inductively Coupled Plasma Optical Emission Spectrometry (ICP-OES) data was obtained with a Agilent 700 Series ICP-OES instrument. Electron Paramagnetic Resonance (EPR) spectra were recorded at 10 K on a Bruker Elexsys E500 spectrometer operating at X band (9.3993 GHz) equipped with a SHQ cavity. A modulation of the magnetic field at 100 kHz with amplitude of 1 G was applied to detect the absorption first derivative. The microwave power was kept at suitable low values (20 mW) to avoid saturation effects. Magnetic measurement: Magnetic susceptibility was measured with a Quantum Design vibrating sample magnetometer. The temperature dependence of the magnetization was measured with 9mg of solid at 5 kOe and corrected from the diamagnetism of the sample and the sample holder. Local order and electronic structure characterizations at the Ti and Fe K edges were carried out at the ROCK beamline (SOLEIL, Gif-sur-Yvette) using the Si(111) channel-cut quick-EXAFS monochromator with an oscillation frequency of 2Hz. Higher harmonics were rejected by using two mirrors coated with B_4C at a grazing incidence of 4 mrad for Ti K edge and 3 mrad for Fe K edge. Pure MIL-177-HT and Fe-doped MIL-177-HT were prepared as pellets diluted with boron nitride which were placed in a liquid nitrogen cryostat. Measurements were carried out at 77K in transmission mode and the EXAFS signal was extracted from a merge of 1000 spectra, each collected in 0.25 s.

Preparation of MIL-177-LT MIL-177-LT was prepared by refluxing H_4mdip linker with Ti(iPrO)$_4$ precursor in formic acid. Solvothermal reactions with the same starting material components in autoclave and glass vial were also carried out for comparison. It was found that no obvious distinction between the products yielded under different synthetic conditions. In clear contrast to fabrication processes of the other reported Ti-MOFs, the condition for MIL-177-LT synthesis not only avoid toxic and complicated reaction systems (such as DMF, HF, additives, prepared cluster precursor, inert atmosphere), but also features easily-handled mild reflux under atmospheric pressure, which is vital for scalable industrial production.

Solvothermal condition in autoclave: To a 23 mL Teflon reactor, H_4mdip (100 mg, 0.29 mmol) and formic acid (5 mL) were added and stirred at room temperature. Ti(iPrO)$_4$ (200 µL, 0.66 mmol) was added dropwise, avoiding forming large pieces of white precipitate. The mixture was kept stirring at room temperature for 30 minutes. And then it was sealed in an autoclave and heated in an oven for 72 hours at 120 °C. After cooled to room temperature, the white solid product was filtered with reduced pressure and washed with EtOH.

Solvothermal condition in glass vial: To a 25 mL glass vial, H_4mdip (100 mg, 0.29 mmol), Ti(iPrO)$_4$ (200 µL, 0.66 mmol) and formic acid (5 mL) were added successively and stirred at room temperature for 30 minutes. Then the vial was tightly sealed with cap and was heated in an oven at 100 °C for 72 hours.

Procedure for PTh isolation MIL-177⊃PTh (82 mg) was vigorously stirred for 2 days in a 0.05 M aqueous solution (100 mL) of sodium ethylenediaminetetraacetate (Na-EDTA) for the removal of the ligand in MIL-177. Subsequently, the solids were collected by centrifugation and washed with HF aqueous solution. The isolated PTh (8 mg) was washed with water and dried under a reduced pressure at room temperature.

Synthesis of Fe doped MIL-177-LT To a 100 mL round bottom flask, H_4mdip (1 g, 2.9 mmol) and formic acid (40 mL) were added and stirred for dispersion at room temperature for 10 minutes. Then $FeCl_3$ anhydrous solid (807 mg, 4.95 mmol, 0.75 equivalents to Ti precursor) was added into the mixture for further stirring at room temperature for another 30 minutes. $Ti(iPrO)_4$ (2 mL, 0.66 mmol) was added at the last step dropwise. Afterwards, the whole reaction mixture was kept reflux for 72 hours. After cooled to room temperature, the light brown solid product was collected by filtration and air-dry followed by washing with water (1L×3) to remove as much as possible the free Fe species.

Preparation of Fe doped MIL-177-HT The same procedure as the one for preparation of pure MIL-177-HT compound was applied.

Detailed conditions for chemical stability tests of MIL-177-LT 20 mg of MIL-177-LT sample was soaking in 100 mL of water at room temperature for 1 month. 20 mg of MIL-177-LT sample was refluxed in boiling water (20 mL) for 24 hours. 50 mg of MIL-177-LT sample was soaking in 20 mL of concentrated HCl (37%) at room temperature for 7 days. 50 mg of MIL-177-LT sample was soaking in 20 mL of concentrated HNO_3 (65%) at room temperature for 7 days. 50 mg of MIL-177-LT sample was soaking in 20 mL of concentrated H_2SO_4 (98%) at room temperature for 7 days. 50 mg of MIL-177-LT sample was soaking in 20 mL of aqua regia at room temperature for 3 days. 50 mg of MIL-177-LT sample was soaking in 20 mL of H_3PO_4 (6M) at room temperature for 7 days. 20 mg of MIL-177-LT sample was soaking in 20 mL of pH = 10 $NaOH/NaHCO_3$ buffer for 24 hours.

Detailed conditions for chemical stability tests of MIL-177-HT 100 mg of MIL-177-HT sample was soaking in 100 mL of water at room temperature for 35 days. 50 mg of MIL-177-HT sample was soaked in hot water (60 °C, 20 mL) for 48 hours. 50 mg of MIL-177-HT sample was soaked

in hot water (70 °C, 20 mL) for 48 hours. 50 mg of MIL-177-HT sample was soaked in hot water (80 °C, 20 mL) for 48 hours. 50 mg of MIL-177-HT sample was soaking in 20 mL of HCl (1 M) at room temperature for 48 hours. 50 mg of MIL-177-HT sample was soaking in 20 mL of HNO_3 (1 M) at room temperature for 48 hours. 50 mg of MIL-177-HT sample was soaking in 20 mL of H_2SO_4 (1 M) at room temperature for 48 hours. 50 mg of MIL-177-HT sample was soaking in 20 mL of H_3PO_4 (0.2 M) at room temperature for 48 hours.

Supplementary References

1. Nevzorov AA. Ergodicity and efficiency of cross-polarization in NMR of static solids. *J Magn Reson* **209**, 161-166 (2011).

2. Saidi F, Taulelle F, Martineau C. Quantitative (13)C Solid-State NMR Spectra by Multiple-Contact Cross-polarization for Drug Delivery: From Active Principles to Excipients and Drug Carriers. *J Pharm Sci* **105**, 2397-2401 (2016).

3. Fung BM, Khitrin AK, Ermolaev K. An improved broadband decoupling sequence for liquid crystals and solids. *J Magn Reson* **142**, 97-101 (2000).

4. Massiot D*, et al.* Modelling one- and two-dimensional solid-state NMR spectra. *Magn Reson Chem* **40**, 70-76 (2002).

5. López R, Gómez R. Band-gap energy estimation from diffuse reflectance measurements on sol–gel and commercial TiO2: a comparative study. *J Sol-Gel Sci Techn* **61**, 1-7 (2011).

6. Krukau AV, Vydrov OA, Izmaylov AF, Scuseria GE. Influence of the exchange screening parameter on the performance of screened hybrid functionals. *J Chem Phys* **125**, 224106 (2006).

7. Vlugt TJH, Krishna R, Smit B. Molecular simulations of adsorption isotherms for linear and branched alkanes and their mixtures in silicalite. *J Phys Chem B* **103**, 1102-1118 (1999).

8. Li W, Walther CF, Kuc A, Heine T. Density Functional Theory and Beyond for Band-Gap Screening: Performance for Transition-Metal Oxides and Dichalcogenides. *J Chem Theory Comput* **9**, 2950-2958 (2013).

9. Foster ME, Azoulay JD, Wong BM, Allendorf MD. Novel metal–organic framework linkers for light harvesting applications. *Chem Sci* **5**, 2081-2090 (2014).

10. Kresse G, Furthmuller J. Efficient iterative schemes for ab initio total-energy calculations using a plane-wave basis set. *Phys Rev B* **54**, 11169-11186 (1996).

11. Hendon CH*, et al.* Engineering the optical response of the titanium-MIL-125 metal-organic framework through ligand functionalization. *J Am Chem Soc* **135**, 10942-10945 (2013).

12. Kresse G, Joubert D. From ultrasoft pseudopotentials to the projector augmented-wave method. *Phys Rev B* **59**, 1758-1775 (1999).

13. Seki S, Saeki A, Sakurai T, Sakamaki D. Charge carrier mobility in organic molecular materials probed by electromagnetic waves. *Phys Chem Chem Phys* **16**, 11093-11113 (2014).

14. Marler B, Oberhagemann U, Vortmann S, Gies H. Influence of the sorbate type on the XRD peak intensities of loaded MCM-41. *Microporous Mater* **6**, 375-383 (1996).

15. Gao L, Li C-YV, Chan K-Y. Polystyrenesulfonate Threaded in MIL-101Cr(III): A Cationic Polyelectrolyte Synthesized Directly into a Metal–Organic Framework. *Chem Mater* **27**, 3601-3608 (2015).

16. Zade SS, Bendikov M. From oligomers to polymer: Convergence in the HOMO-LUMO gaps of conjugated oligomers. *Org Lett* **8**, 5243-5246 (2006).

17. Singh V*, et al.* High thermal conductivity of chain-oriented amorphous polythiophene. *Nat Nanotechnol* **9**, 384-390 (2014).

18. MacLean MW*, et al.* Unraveling Inter- and Intrachain Electronics in Polythiophene Assemblies Mediated by Coordination Nanospaces. *Angew Chem Int Edit* **55**, 708-713 (2016).

19. Wang H*, et al.* Exciton diffusion and charge transfer dynamics in nano phase-separated P3HT/PCBM blend films. *Nanoscale* **3**, 2280-2285 (2011).

20. Wang LJ*, et al.* Synthesis and Characterization of Metal-Organic Framework-74 Containing 2, 4, 6, 8, and 10 Different Metals. *Inorg Chem* **53**, 5881-5883 (2014).

21. Hodeau JL*, et al.* Nine crystal multi-analyser stage for high resolution powder diffraction between 6 and 40keV. (ed^(eds). Crystal and Multilayer Optics (1998).

22. Topas V5: General profile and structure analysis software for powder diffraction data. *Bruxer AXS Ltd*, (2014).

23. Altomare A*, et al.* EXPO: a program for full powder pattern decomposition and crystal structure solution. *J Appl Crystallogr* **32**, 339-340 (1999).

24. Spek AL. Structure validation in chemical crystallography. *Acta Crystallogr D* **65**, 148-155 (2009).

25. VandeVondele J, Krack M, Mohamed F, Parrinello M, Chassaing T, Hutter J. Quickstep: Fast and accurate density functional calculations using a mixed Gaussian and plane waves approach. *Comput Phys Commun* **167**, 103-128 (2005).

26. Hutter J, Iannuzzi M, Schiffmann F, VandeVondele J. cp2k:atomistic simulations of condensed matter systems. *WIRES COMPUT MOL SCI* **4**, 15-25 (2014).

27. Perdew JP*, et al.* Restoring the density-gradient expansion for exchange in solids and surfaces. *Phys Rev Lett* **100**, 136406 (2008).

28. VandeVondele J, Hutter J. Gaussian basis sets for accurate calculations on molecular systems in gas and condensed phases. *J Chem Phys* **127**, 114105 (2007).

29. Goedecker S, Teter M, Hutter J. Separable dual-space Gaussian pseudopotentials. *Phys Rev B* **54**, 1703-1710 (1996).

30. Grimme S. Semiempirical GGA-type density functional constructed with a long-range dispersion correction. *J Comput Chem* **27**, 1787-1799 (2006).

31. Stern EA. NUMBER OF RELEVANT INDEPENDENT POINTS IN X-RAY-ABSORPTION FINE-STRUCTURE SPECTRA. *Phys Rev B* **48**, 9825-9827 (1993).

32. Mazik M, König A. Mimicking the Binding Motifs Found in the Crystal Structures of Protein–Carbohydrate Complexes: An Aromatic Analogue of Serine or Threonine Side Chain Hydroxyl/Main Chain Amide. *Eur J Org Chem* **2007**, 3271-3276 (2007).
