# Supplementary material for: A phase transformable ultrastable titanium-carboxylate framework for photoconduction
Source: Nat Commun. 2018 Apr 25;9:1660. doi: 10.1038/s41467-018-04034-w (PMC5916937; doi:10.1038/s41467-018-04034-w)
Supplement: Supplementary file 3 — Description of Additional Supplementary Files [file 41467_2018_4034_MOESM3_ESM.pdf]

Description of Additional Supplementary Files

File Name: Supplementary Data 1
Description: CIF file of MIL-177-LT.

File Name: Supplementary Data 2
Description: CIF file of MIL-177-HT.
